# Meta-Analysis of Brain Volumetric Abnormalities in Patients with Remitted Major Depressive Disorder

**DOI:** 10.1155/2024/6633510

**Published:** 2024-05-15

**Authors:** Xin Xu, Qian Zhou, Fei Wen, Mingzhe Yang

**Affiliations:** Department of Psychology, The Second Affiliated Hospital of Guangzhou Medical University, Guangzhou, China

## Abstract

Although patients with major depressive disorder (MDD) achieve remission after antidepressant treatment, >90% of those in remission have at least one residual depressive symptom, which may be due to neural damage linked with MDD. To better understand the structural impairments in patients with remitted MDD, we conducted a meta-analysis comparing grey matter volume (GMV) abnormalities between patients with remitted MDD and healthy controls (HCs). There were 11 cross-sectional datasets that investigated 275 patients with remitted MDD versus 437 HCs, and 7 longitudinal datasets that investigated 167 patients with remitted MDD. We found that GMV in the left insula, inferior parietal gyri, amygdala, and right superior parietal gyrus was decreased in patients with remitted MDD than in HCs. Additionally, patients with remitted MDD had lower GMV in the bilateral gyrus rectus than those in the nonremission state. Moreover, increased GMV in the bilateral anterior cingulate cortex, right striatum, middle temporal gyrus, and superior frontal gyrus was observed in patients with remitted MDD than in HCs. Furthermore, patients with remitted MDD had a larger GMV in the bilateral median cingulate/paracingulate gyri, left striatum, putamen, amygdala, hippocampus, and parahippocampal gyrus at follow-up than at baseline. Based on the brain morphological abnormalities in patients with remitted MDD after electroconvulsive therapy and pharmacological treatment, we proposed a schematic diagram of targeted intervention approaches for residual symptoms. In summary, our findings provide neurobiology-based evidence for multitarget treatment of depression to reduce residual symptoms and improve social function in patients with MDD.

## 1. Introduction

Major depressive disorder (MDD) is a clinical syndrome with depressed mood and anhedonia as its core symptoms. MDD leads to a severe disease burden due to its high recurrence rate and impairment of psychosocial function [1, 2]. Although patients with MDD achieve remission after antidepressant treatment, >90% have at least one residual depressive symptom [3, 4], such as negative affective cognition, attention deficit [5], and negative reward expectation [4, 6]. Moreover, 20% of patients with remitted MDD experience a relapse of depression within 6 months of taking antidepressants [7]. Posttreatment residual symptoms are prognostic risk indicators [8] and precursors of depression recurrence [9]. Neuroimaging studies have found that patients with remitted MDD have brain structural abnormalities [10, 11] and neural activity dysfunction [12, 13], which are linked with residual symptoms, compared to healthy controls (HCs). Exploring brain morphological abnormalities in patients with remitted MDD would help better understand the neurobiological basis of residual symptoms of depression to achieve targeted intervention suitable for the residual symptom spectrum.

Previous meta-analyses confirmed changes in grey matter volume (GMV) in patients with depressive episodes [14–16]; however, volumetric changes in patients with remitted MDD remain unclear. Although multiple neuroimaging studies have shown brain abnormalities in patients with remitted MDD compared to HCs [11, 17, 18], these results are inconsistent. For example, one study showed that GMV in the right superior temporal gyrus (STG) was increased in patients with remitted MDD than in HCs [18]. In another study, patients with both remitted and current MDD showed reduced GMV in the left insula compared with that in HCs [17]. A possible reason for this is that antidepressants are only effective in the brain regions where the target neurotransmitter receptors are distributed, and antidepressants do not completely ameliorate all damaged brain regions. A previous meta-analysis reported persistent brain dysfunction in patients with remitted MDD [13]; however, no meta-analysis of volumetric changes in these patients has been reported.

To better understand the structural abnormalities in remitted MDD, we performed a meta-analysis of differences in GMV between patients with remitted MDD and HCs using the anisotropic effect size version of signed differential mapping (AES-SDM). SDM can incorporate negative results and has been used in several neuroimaging meta-analyses [15, 19]. Based on the results of the meta-analysis, we created a diagram of residual symptoms linked with brain morphological abnormalities in patients with remitted MDD and possible interventions to select appropriate treatment modalities according to neurobiological evidence to prevent depression recurrence in the future.

## 2. Methods

### 2.1. Literature Search

To identify relevant structural articles focused on patients with remitted MDD, two investigators independently conducted comprehensive literature searches using PubMed, EBSCOhost, and ProQuest through October 2023, following the Preferred Reporting Items for Systematic reviews and Meta-Analyses guidelines [20]. Search keywords included (1) “depressive disorder” or “unipolar depression” or “depression” or “depress∗” plus (2) “VBM” or “voxel” or “voxel-based morphometry” or “morphometry” plus (3) “remit∗” or “remission” or “remitted” or “recover∗” or “recovery”. The language was limited to English.

### 2.2. Meta-Analysis Selection Criteria

Two researchers independently screened the studies according to the inclusion criteria. If there was any inconsistency in study selection, a consensus was reached though discussion.

The inclusion criteria were as follows: (1) studies investigating adults diagnosed with MDD according to the Diagnostic and Statistical Manual of Mental Disorders or International Classification of Diseases, Tenth Revision criteria; (2) cross-sectional studies comparing patients with remitted MDD with HCs, and longitudinal studies should compare baseline and follow-up data of patients with remitted MDD; (3) grey matter research using whole-brain voxel-based morphometry (VBM); (4) studies with patients aged 18-70 years; (5) studies reporting coordinates in a standard space such as the Talairach space or the Montreal Neurological Institute (MNI) space.

Patients with remitted MDD were defined by a 17-item Hamilton Depression Rating Scale (HDRS-17) or HDRS-24 score of ≤7, a Beck Depression Inventory (BDI) score of ≤14, or a Montgomery-Åsberg Depression Rating Scale (MADRS) score of ≤6 [21, 22].

The exclusion criteria were studies (1) wherein coordinates were not clearly reported; (2) that did not use VBM; (3) wherein patients had a history of alcohol or substance abuse, head trauma, and major physical or neurological illness; and (4) wherein patients had a comorbidity of schizophrenia, bipolar disorders, other major psychoses, obsessive-compulsive spectrum disorders, posttraumatic stress disorder, or cluster B personality disorders.

### 2.3. Data Extraction for Systematic Review

The first author's name, year of publication, details of study design, patient characteristics (including gender, age, age of onset, illness duration, number of episodes, comorbidity with anxiety disorder, and disease severity), sample size, magnetic resonance imaging (MRI) sequence acquisition parameters, and changes in VBM were collected from the eligible studies. From each included study, we selected the reported peak coordinates of GMV differences and *t*-value threshold that were statistically significant at the whole-brain level. The number of patients in the longitudinal study was the number of patients with MDD who completed the second scan, and age, age at onset, illness duration, number of episodes, and comorbidity rate with anxiety were considered baseline data.

### 2.4. SDM Analysis

A meta-analysis of regional GMV abnormalities was conducted using AES-SDM (https://www.sdmproject.com/). AES-SDM performs voxel-wise random effects meta-analyses by reconstructing whole-brain effect size and variance maps that combine the original statistical parametric maps and peak coordinates from both positive and negative results [19]. However, including negative effects could reduce the risk of a particular voxel showing opposite effects. First, pooled analyses were conducted to investigate regional GMV differences within the remitted MDD group compared to the HC group. To obtain more accurate results, we defined the *p* value threshold in the AES-SDM analysis as <0.05 and only discussed brain regions with voxels as >10 voxels. Whole-brain jack-knife sensitivity analyses were conducted to investigate the overlap between significant areas of heterogeneity with areas of grey matter differences. Separate simple metaregressions were performed by using available potential confounders provided in a sufficient proportion of the included studies. Figures were prepared using MRIcron and BrainNetViewer.

## 3. Results

### 3.1. Literature Search and Sample Characteristics

**Figure 1:**
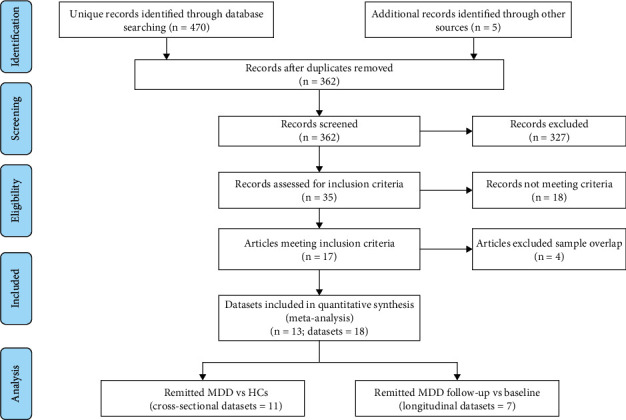
Flow chart of literature search and selection in the meta-analysis. Abbreviations: HCs: healthy controls; MDD: major depressive disorder; *n*: number.

A flow diagram of the study selection process is shown in Figure 1. As shown in Table 1, the meta-analysis of the cross-sectional studies included 11 whole-brain VBM studies [17, 18, 22–30], among which negative results were obtained. Two of them were after electroconvulsive therapy (ECT) [18, 30], and the other after antidepressant treatment. There were 11 datasets that investigated 275 patients with remitted MDD (182 women; mean age 43.57 years) versus 437 HCs (281 women; mean age 41.51 years). As shown in Table 2, the meta-analysis of longitudinal studies included 7 whole-brain VBM datasets [23–26, 30–32] that investigated 167 patients with remitted MDD (women 97; mean age 42.97 years). Of these, two had a follow-up period of > two years, and five had a follow-up period of ≤8 weeks.

### 3.2. GMV in Patients with Remitted MDD Compared with HC

**Figure 2:**
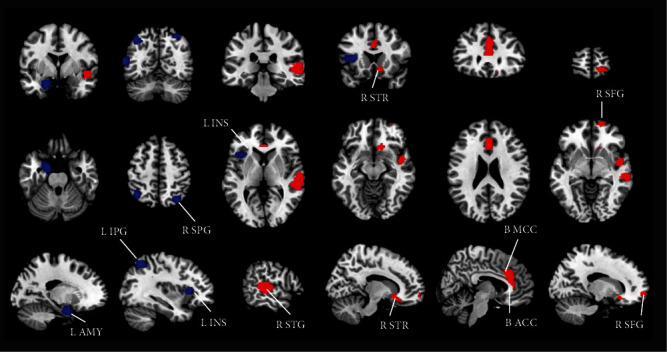
Meta-analysis results of GMV difference in patients with remitted MDD compared with HCs. The areas of decreased GMV compared with HC are displayed in blue, and the areas of increased GMV are displayed in red. Abbreviations: ACC: anterior cingulate cortex; AMY: amygdala; GMV: grey matter volumes; HCs: healthy controls; IFG: inferior frontal gyrus; INS: insula; IPG: inferior parietal gyri; L: left; MCC: median cingulate cortex; MDD: major depressive disorder; MTG: middle temporal gyrus; R: right; SPG: superior parietal gyrus; STG: superior temporal gyrus; STR: striatum.

As shown in Table 2 and Figure 2, patients with remitted MDD had a larger GMV in the bilateral median cingulate/paracingulate cortex (MCC), anterior cingulate/paracingulate cortex (ACC), right STG extending to the temporal pole, middle temporal gyrus (MTG), superior frontal gyrus (SFG) orbital part, and striatum than that in HCs. Compared to that in HC, patients with remitted MDD had a lower GMV in the left insula, MTG, inferior frontal gyrus (IFG), inferior parietal gyrus (IPG), parahippocampal gyrus, amygdala, hippocampus, and right superior parietal gyrus (SPG).

### 3.3. GMV Changes in Patients with Remitted MDD in Longitudinal Studies

**Figure 3:**
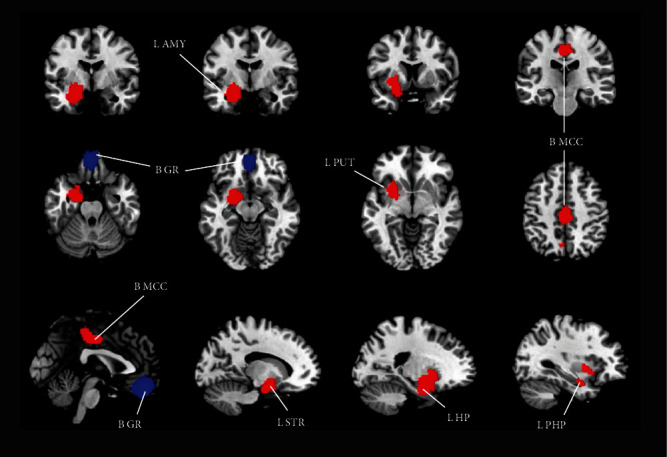
GMV changes in patients with remitted MDD in longitudinal studies. The areas of lower GMV in patients with remitted MDD than that of those in the nonremission state are displayed in blue, and the areas of increased GMV are displayed in red. Abbreviations: AMY: amygdala; GMV: grey matter volumes; GR: gyrus rectus; HCs: healthy controls; HP: hippocampus; L: left; MCC: median cingulate cortex; MDD: major depressive disorder; PHP: parahippocampal gyrus; PUT: putamen; R: right; STR: striatum.

As shown in Table 3 and Figure 3, patients with remitted MDD had a larger GMV in the bilateral MCC, left striatum, putamen, amygdala, hippocampus, and parahippocampal gyrus at follow-up than at baseline. In contrast, the GMV in the bilateral gyrus rectus decreased from baseline to follow-up.

### 3.4. Subgroup Analysis and Metaregression Analysis

Subgroup analysis of the studies after pharmacological treatment is shown in Table S1 and Figure S1. Excluding the ECT study, patients with remitted MDD had a larger GMV in the bilateral ACC, right striatum, gyrus rectus, and left IFG than that in HCs. Compared to that in HC, patients with remitted MDD showed reduced GMV in the left MTG, insula, IPG, and right SPG after antidepressant treatment.

Metaregression analysis did not find significant correlations between MDD-related GMV changes and age, age of onset, female percentage, the number of depressive episodes, comorbidity rate with anxiety, severity of depressive symptoms, or duration of illness.

### 3.5. Jack-Knife Sensitivity Analyses and Publication Bias Analysis

Whole-brain jack-knife sensitivity analyses of the cross-sectional studies revealed an increased GMV in the right striatum in all 11 analyses (Table S2 in the supplement). Increased GMV in the bilateral ACC and decreased GMV in the left IPG and right SPG were observed in 10 analyses. Other abnormal brain areas remained replicable and were found in at least 9 studies. In subgroup analysis, decreased GMV in the right SPG and increased GMV in the right striatum were observed in all 9 analyses (Table S3 in the supplement). Other abnormal brain areas remained replicable which were found in at least 9 studies.

Whole-brain jack-knife sensitivity analyses of the longitudinal studies revealed an increased GMV in the bilateral MCC and left striatum in 6 analyses (Table S4 in the supplement). Decreased GMV in the bilateral gyrus rectus and left SPG were observed in 6 analyses. Other abnormal brain areas remained replicable and were found in at least 5 studies (Table S4 in the supplement).

As shown in Figure S2 and Figure S3, an analysis of publication bias of the cross-sectional studies detected by funnel plot asymmetry revealed that all Egger's tests of publication bias were nonsignificant (all *p* values > 0.05) except in the left insula (*p* = 0.027). As shown in Figure S4, Egger's tests of publication bias of the longitudinal studies were nonsignificant in the bilateral MCC and left striatum (all *p* values > 0.05).

## 4. Discussion

To the best of our knowledge, the current meta-analysis is the first to identify GMV abnormalities in patients with remitted MDD compared with HCs. We found that patients with remitted MDD had decreased GMV in the left insula, IPG, and right SPG compared with that in HCs. Additionally, the GMV in the bilateral gyrus rectus in patients with remitted MDD was lower at follow-up than at baseline. In contrast, the GMV increased in bilateral ACC and MCC, right striatum, MTG, and STG in patients with remitted MDD compared with that in HCs. Moreover, patients with remitted MDD had a larger GMV in the bilateral MCC, left striatum, putamen, amygdala, hippocampus, and parahippocampal gyrus at follow-up than at baseline. Among the studies included in this meta-analysis, patients with remitted MDD received only ECT and medications. However, these therapeutic approaches do not fully cover all lesions or pathophysiological pathways. Furthermore, we proposed a schematic diagram of the targeted intervention approaches for residual symptoms, according to the brain morphological abnormalities in patients with remitted MDD after ECT and medical therapy.

### 4.1. Decreased GMV in the Neocortex in Patients with Remitted MDD

Our results showed that patients with remitted MDD had decreased GMV in the left insula, IPG, and right SPG compared with that in HCs. The left insula, IPG, and right SPG all belong to the neocortex, which processes complex cognition [33–35]. Previous meta-analyses have also found extensive cortical GMV reduction in the insular, prefrontal, and parietal regions in patients with MDD [15]. Micropathological changes that contribute to reduced GMV in patients with MDD include atrophy of neurons, dendritic cells, and glial cells [36], while the molecular mechanisms involve increased expression of inflammatory mediators, mitochondrial dysfunction, and decreased levels of brain-derived neurotrophic factor (BDNF) [37, 38]. Structural MRI studies have shown that a high level of peripheral inflammatory markers correlates with reduced GMV in the neocortex of patients with MDD [39, 40]. Both oxidative stress and reduced ATP production contribute to nerve cell regeneration damage, mitochondrial dysfunction, and changes in neuroplasticity [41, 42].

Both clinical and animal experiments have shown that decreased GMV in patients with MDD improves with symptom remission. A longitudinal study found that whole-brain cortical thickness increased in patients with MDD after remission compared to that before treatment [10]. Animal studies have shown that depression-like symptoms in rats are relieved by an increase in the number of frontal cortex cells and an extension of dendritic length [43], suggesting that dendritic cell atrophy could be reversible. Animal studies have shown that selective serotonin reuptake inhibitors (SSRIs), such as fluoxetine, can inhibit apoptotic kinase activation, increase BNDF levels, and reverse astrocytic atrophy [44], suggesting that the decrease in GMV caused by astrocyte atrophy is reversible. In contrast, previous postmortem studies have reported that patients with MDD who died from suicide showed reduced pyramidal neuron density in the neocortex, implying apoptosis of pyramidal neurons [45]. Neurogenesis in adults occurs in the hippocampus but not in the neocortex [46, 47]. In other words, GMV reduction caused by the atrophy of dendritic cells and astrocytes is reversible, but that caused by the apoptosis of neocortical pyramidal neurons is not.

#### 4.1.1. GMV in the Left Insula in Patients with Remitted MDD

This study demonstrated that the GMV in the left insula was lower in patients with remitted MDD than that in HCs. This is consistent with the results of a previous study that reported that the cortical thickness of the insula is significantly thinner in remitted MDD patents than in HCs [48]. Previous studies have found that GMV reduction in the left insula correlates with depression severity and illness duration in patients with MDD [49, 50]. Longitudinal studies have found that the cortical thickness of the insula in patients was significantly greater in the remission group than before treatment [10] and in the nonremission group [51] after antidepressant treatment. After ECT, patients with remitted MDD also showed increased cortical thickness and surface area in the left insula compared with those before treatment [52]. This neurobiological evidence suggests that the GMV in the insula of patients with remitted MDD remains lower than that in HCs, even if medications and ECT can ameliorate the loss of GMV caused by depressive episodes.

Patients with remitted MDD have persistent negative attention bias and bradykinesia, which are linked to the dysfunction of the insula [53]. The insula is involved in self-perception and self-worth judgments [54], and its impairment can lead to self-perception disorders such as negative self-evaluation [55], guilt and shame/embarrassment [56, 57], borderline personality disorder [58], and suicide [59], which are risk factors for depression recurrence [60, 61]. A previous study reported that cognitive behavioral therapy could weaken insular activity during emotion perception [62]. A meta-analysis showed that insula activation in patients with MDD was significantly reduced after psychotherapy [63], suggesting that psychotherapy could be effective in improving the residual self-cognitive impairment of depression in remission.

#### 4.1.2. GMV in the Frontal-Parietal Attention Network in Patients with Remitted MDD

Compared with HCs, patients with remitted MDD showed reduced GMV in the left IPG and right SPG. This result is consistent with previous multicenter research that showed reduced cortical thickness in the parietal lobe in patients with current MDD [64]. The left IPG and right SPG belong to the frontoparietal attention network [65], which is involved in selective attention [66, 67] and execution functions [68, 69]. Reduced activation in bilateral IFG in patients with remitted MDD is linked to cognitive reappraisal ability impairment for attention goals [70]. Abnormal functional activity in the frontoparietal network is correlated with executive dysfunction and working memory in patients with remitted MDD [71–73].

The bilateral dorsolateral prefrontal cortex (DLPFC) is a key node of the frontoparietal attention network [74]. Transcranial magnetic stimulation (TMS) of the DLPFC can regulate brain activity in the parietal nodes of the frontoparietal attention network through interactions between brain networks [75, 76] and has been recommended as a treatment for MDD [77]. Previous studies have shown that repetitive TMS (rTMS) could improve attention [78], working memory [79], executive function [80], and cognitive control [81] in patients with MDD. Additionally, functional activity in the parietal cortex is useful for predicting TMS outcomes in patients [82]. Thus, patients with remitted MDD require rTMS therapy if they have residual attention and executive dysfunction.

#### 4.1.3. GMV of the Gyrus Rectus in Patients with Remitted MDD Was Lower than Those without Remission

Our study suggested that GMV in the gyrus rectus was reduced from baseline to follow-up with SSRI treatment, suggesting that 5-HT-ergic drugs were ineffective for the gyrus rectus. As part of the anterior cingulate gyrus extending into the frontal lobe, the gyrus rectus receives projections from the hypothalamus and brain stem and is involved in sensory integration [83]. The expression of the 5-HT(2A) receptor in the gyrus rectus decreased with increasing age starting at 20 years [84]. Therefore, the nonresponse of the gyrus rectus to SSRIs may be due to the insufficient distribution of 5-HT(2A) receptors. Compared to that in HCs, reduced GMV in the gyrus rectus was reported in patients with MDD, schizophrenia, and bipolar disorder [85, 86], suggesting that the decreased GMV may have a more complex pathological basis. Research using deep brain stimulation revealed that the gyrus rectus is an effective target for treatment-resistant depression [87]. Neuroregulatory therapies (TMS, DBS) can target cortical sites [77, 88, 89], and SSRIs mainly act on subcortical structures along the distribution of 5-HT-energy receptors [3]. However, both have their own advantages. If a patient shows signs of neocortical impairment, future treatment should consider neuroregulatory therapy such as TMS as an equally important first-line treatment along with drugs rather than as a second-line treatment only for treatment-resistant depression.

### 4.2. Larger Reward Circuit GMV in Patients with Remitted MDD than in HCs and at Baseline

Our meta-analysis showed that patients with remitted MDD had greater GMV in the striatum than in HCs and at baseline. However, reduced striatal GMV in medication-naïve patients with first-episode MDD was found in previous meta-analyses [14, 16]. Clinical studies have also found an increase in striatal GMV in patients with MDD after ECT [31]. Machine learning studies have demonstrated that morphological changes in the ventral striatum predict improvements in serotonin and norepinephrine reuptake inhibitors [90]. Therefore, elevated GMV in the striatum may indicate relief of depressive symptoms in patients with MDD.

Moreover, the meta-analysis of cross-sectional studies found that patients with remitted MDD had greater GMV in bilateral ACC and MCC than that in HCs, and the meta-analysis of longitudinal studies found that patients with remitted MDD had greater GMV in bilateral MCC than before remission. Notably, increased cortical thickness in ACC has also been observed in unmedicated patients with first-episode MDD [91]. One possible explanation for this phenomenon is that the GMV increases due to activated microglia and reactive astrogliosis [92, 93] when the activated immune response system (IRS) and compensatory IRS (CIRS) pathway were elevated during the acute phase of depressive episodes [94, 95]. However, the sensitive IRS and CIRS responses did not return to homeostasis after the remission of MDD [96]. Thus, increased GMV of the ACC in patients with remitted MDD may indicate an active compensatory immune response.

The increase in GMV in the reward circuit does not equate to the complete recovery of function. Both the striatum and ACC are core nodes of the reward circuit [97] and participate in the encoding and processing of reward anticipation information. Damage to the reward circuit contributes to anhedonia [98], which is a core symptom of major depressive disorder. The response of the corticostriatal network is still lower in patients with remitted MDD when they perform the reward expectation prediction task [6]. When individuals in remission of depression perform the reward prediction error task, the functional activity of the bilateral striatum is lower than that of the HC group [99], which also indicates that individuals in remission of depression still have a negative cognitive model of reward expectation despite the improvement of depressive mood. Compared to HCs, the functional activation of the striatum was increased in the patients with remitted MDD during the execution of stressful tasks [100], implying that patients with remitted MDD need to expend more energy to solve stress problems with worse stress resistance.

Dysfunction of the reward circuits is highly correlated with inflammation [101]. The low response of the ventral striatum to a rewards-anticipation task is related to a high level of peripheral blood leukocyte reactivity in patients with depression [102]. Functional connectivity between the striatum and ventromedial prefrontal cortex (vmPFC) mediates peripheral C-reactive protein (CRP) levels and anhedonia [103]. However, clinical trials have shown that anhedonia is negatively correlated with the strength of functional connectivity between the striatum and vmPFC only in patients with CRP levels > 2 mg/L [98], suggesting that examination of inflammation-related indicators in patients is necessary to distinguish depression subtypes and choose the appropriate treatment [104]. Although there is a lack of neuroimaging evidence for the effects of anti-inflammatory drugs on MDD, clinical studies have shown that anti-inflammatory drugs, such as nonsteroidal anti-inflammatory drugs, omega-3 fatty acids, and statins, are effective in improving depressive symptoms [105].

### 4.3. GMV Abnormalities in the Right Temporal Lobe in Patients with Remitted MDD

The pooled meta-analysis revealed that patients with remitted MDD showed increased GMV in the right MTG and STG extending to the temporal pole compared with that in HCs, but the results of the subgroup analysis did not show these abnormalities after excluding the ECT study. A previous study reported that both patients with current and remitted MDD showed decreased GMV in the right STG relative to that in HCs and that the GMV of the right STG was associated with the severity of depressive symptoms [106]. Another study found that the thickness of the temporal pole and insular cortex increased after ECT compared to that before ECT [107], suggesting that the increase in GMV in the temporal lobe might be an effect of ECT. Additionally, a meta-analysis also reported increased GMV in the right medial temporal lobe, the amygdala, and the hippocampus in patients with multiple mental disorders after ECT [108, 109].

GMV in the right STG was positively correlated with rumination in patients with MDD [110]. Additionally, trauma in patients with MDD is negatively correlated with the cortex thickness of the left MTG [111] and bilateral MTG activity [112]. Brain activity in the right MTG was also reportedly reduced in patients with remitted MDD [113]. Moreover, behavioral experiments have confirmed that rumination is positively correlated with auditory hallucination [114], and neuroimaging studies have revealed that rumination and auditory hallucinations were both correlated with structural and functional abnormalities of the STG [115, 116]. This suggests that improving the local neural activity of the STG may ameliorate rumination. Given that low-frequency temporoparietal junction- (TPJ-) TMS has been successfully used to treat auditory hallucinations [117], we hypothesized that TPJ-TMS would be equally effective against rumination, even though current neuromodulation studies all reported the stimulation target for rumination was the left DLPFC [118, 119].

This meta-analysis of longitudinal studies revealed that GMV increased in the hippocampus, parahippocampal gyrus, and amygdala in patients with remitted MDD than at baseline, whereas the meta-analysis of cross-sectional studies showed decreased GMV in the hippocampus, parahippocampal gyrus, and amygdala in patients with remitted MDD compared to HCs, suggesting that patients did not fully return to normal despite treatment response. The amygdala is involved in emotional responses and is easily overactivated by negative emotional stimuli in the depressed state, corresponding to negative emotional sensitivity in patients with MDD [120]. SSRI treatment normalizes the overactivation of the amygdala to negative stimuli [62, 121]. The hippocampus is densely innervated by 5-hydroxytryptaminergic fibers [122], and neurogenic dysregulation of the dentate gyrus in the hippocampus occurs in MDD [123]. Animal studies have shown that SSRIs not only promote cell proliferation and differentiation in the hippocampus [124] but also affect gamma-aminobutyric acid and glutaminergic neurotransmission [125]. Therefore, increased GMV in the hippocampus, parahippocampal gyrus, and amygdala from baseline to remission is an imaging feature of improvement in depression.

### 4.4. Schematic Diagram of Brain Morphological Abnormalities Linked with Residual Symptoms in Patients with Remitted MDD

**Figure 4:**
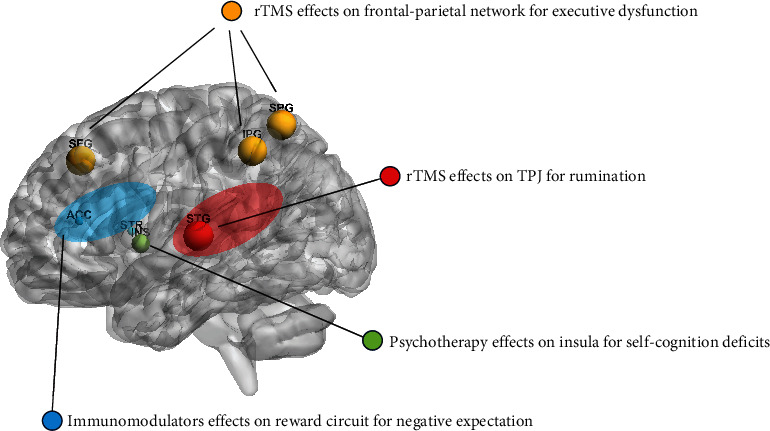
Brain location of novel and emerging treatment for residual symptoms in patients with remitted MDD. Abbreviations: ACC: anterior cingulate cortex; INS: insula; IPG: inferior parietal gyri; MDD: major depressive disorder; rTMS: repetitive transcranial magnetic stimulation; SFG: superior frontal gyrus; SPG: superior parietal gyrus; STG: superior temporal gyrus; STR: striatum; TPJ: temporoparietal junction.

Figure 4 shows a schematic diagram of possible treatments for residual symptoms of depression. Treatment for residual symptoms of depression mostly involves psychotherapy [126, 127], while clinical guidelines recommend rTMS for treatment-resistant depression [77]. Considering the presence of GMV abnormalities in patients with remitted MDD, rTMS has a potential therapeutic value for residual symptoms of depression. Here, we list the potential interventions for residual symptoms, including negative executive dysfunction, rumination, self-perception, and negative reward anticipation [4, 6, 8, 99], which are located in the key hub of related neural networks, according to GMV abnormalities in patients with remitted MDD.

Executive dysfunction is associated with dysfunction of the frontal-parietal network [74], and TMS of the DLPFC can improve executive dysfunction [76, 80]. The rTMS of the precuneus has been performed to improve working memory [128], suggesting that the parietal nodes of the frontoparietal network may also be a novel target for improving executive function.

Rumination is correlated with impairment of the temporal lobe in patients with depression, and low-frequency rTMS of the TPJ is a potential target for rumination treatment [117, 118].

Negative self-perceptions, such as self-blame and self-guilt, are linked to insular impairment, and insular dysfunction in patients is significantly improved after psychotherapy [63]. Thus, psychotherapy for negative self-cognition is still needed, even after the remission of depression.

The negative reward expectation is associated with impairment of the reward circuit in patients with remitted MDD [99, 100]. The efficacy of immunomodulation in depressive disorders has been confirmed in clinical trials, despite a lack of direct neuroimaging evidence [105, 129].

### 4.5. Limitations

This study has some limitations. First, the meta-analysis of cross-sectional studies included only 11 datasets, and the meta-analysis of longitudinal studies included only 7 datasets. To reduce the heterogeneity of the data analysis methods, we only extracted the results of the VBM studies, which may have limited the number of included studies. A previous meta-analysis for the fMRI features of remitted MDD included 18 datasets [13], in which the number of fMRI studies was higher than that of VBM studies. A possible reason is that brain structural changes are not obvious after a short duration of treatment. The minimum duration of depression remission included in this review was 4 months [25], which was different from that in most functional imaging studies (1-2 months) [13].

In addition, there was a lack of information on the duration of remission in the included studies, and a longitudinal comparison from the acute phase to symptom remission was added in the meta-analyses of longitudinal studies, in which only Lemke et al. included patients with lower mean HDRS scores at baseline. However, patients with acute phase episodes remained in the baseline group.

Moreover, because a meta-analysis of GMV in patients with current MDD has been conducted previously [14–16], this was not performed in the present study. This meta-analysis included the negative results that no significant GMV abnormalities between patients with remitted MDD and HCs were reported in two studies that recruited patients with illness duration lasting more than 10 years [22, 29]. Thus, the influence of illnesses cannot be ignored, even though the regression analysis is inconclusive. Notably, one study reported a significant change in grey matter density but not in GMV in patients with remitted MDD [22], suggesting that only one indicator is limited.

Although the search strategy we used did not limit the treatment modalities, only medication and ECT were searched in the included studies of GMV changes in this population. Two researchers independently searched for neuroimaging studies associated with depression remission after psychotherapy but only found fMRI studies that reported that improvements in depressive symptoms after psychotherapy were associated with changes in the functional activity of the prefrontal and limbic cortices [63, 130, 131]. Although the results of this study were dominated by drug- and ECT-induced changes in GMV, we also discussed the therapeutic potential of psychotherapy for residual symptoms of depression.

Additionally, this study only discussed residual symptoms that may be associated with the abnormal brain areas found in the current meta-analysis and did not review all residual symptoms. Some residual symptoms involving extracerebral systems, such as tension involving overactivity of the hypothalamic-pituitary-adrenal axis, should be concentrated in the future [132].

Finally, owing to the inherent limitations of meta-analysis, the possibility of publication bias cannot be completely ruled out, despite our best efforts to search for more original and appropriate literature, including studies with negative outcomes.

## 5. Conclusion

Overall, this meta-analysis demonstrated that patients with remitted MDD exhibited reduced GMV in the insula and frontal-parietal network and increased GMV in the reward circuit and temporal lobe after receiving medications and undergoing ECT. Our findings provide new insights into targeted treatment for residual symptoms based on neurobiology-based evidence, combined with anti-inflammatory medication, TMS, psychotherapy, and other treatment modalities.

## Figures and Tables

**Table 1:** Sample characteristics and MRI procedures of included cross-sectional studies.

Study (publication year)	Number (female)		Mean age (y)		Age of onset	Illness duration (months)	No. of episodes	Mean depression severity	Therapy	Comorbidity with anxiety (rate%)	MRI procedures		
	MDD	HC	MDD	HCs							Field strength	Voxel size (mm^3^)	MRI scanner
Arnone et al. (2013) [23]	25 (20)	66 (46)	34.5	32.1	22.0	NA	2.8	2.2 (MADRS)	Medication-free	0/23 (0%)	1.5 T	0.875∗0.875∗1	Philips Intera
Fang et al. (2015) [24]	20 (8)	18 (8)	59.2	59.1	56.1	43.2	2.1	6.4 (HDRS-17)	Antidepressants	NA	1.5 T	1∗1∗1	Siemens
Klauser et al. (2015) [22]	27 (18)	33 (21)	35.0	34.7	10.29	137.4	3.1	13.0 (BDI)	Antidepressants	22/56 (39%)	1.5 T	1∗1∗1	Siemens
Kong et al. (2014) [25]	24 (14)	28 (14)	36.1	32.1	NA	4.12	NA	3.4 (HDRS)	Fluoxetine	NA	1.5 T	1.5∗1.5∗1.5	GE Signa
Lemke et al. (2022) [26]	42 (24)	63 (39)	38.79	36.48	NA	14.06	NA	2.55 (HDRS)	Antidepressants	NA	3.0 T	NA	NA
Li et al. (2010) [27]	19 (13)	25 (19)	42.6	40.6	36.5	112.8	3.5	3.4 (HDRS)	SSRI/SNRI/bupropion	13/44 (30%)	1.5 T	2∗2∗2	NA
Liu et al. (2014) [17]	19 (19)	19 (19)	37.6	36.8	NA	88.44	2.39	4.6 (HDRS)	Antidepressants	NA	1.5 T	1∗1∗1.33	Siemens Trio
Salvadore et al. (2011) [28]	27 (21)	107 (60)	40.2	36.2	NA	181.2	NA	1.6 (MADRS)	Medication-free	NA	3.0 T	3∗3∗3	Milwaukee
Serra-Blasco et al. (2013) [29]	22 (20)	32 (23)	48	46.0	43.5	214.3	4.56	4.0 (HDRS)	Antidepressants	NA	3.0 T	NA	Philips Achieva
Takamiya et al. (2021) [18]	27 (13)	21 (19)	67.5	63.0	NA	60	NA	6.0 (HDRS-17)	MECT+antidepressant+antipsychotics+benzodiazepine	NA	3.0 T	0.9∗0.9∗1.0	GE Signa HDxt
Wang et al. (2017) [30]	23 (12)	25 (13)	38.74	39.52	33.90	70.35	NA	3.83 (HDRS-17)	MECT+antidepressant+antipsychotics	NA	3.0 T	3.4∗3.4∗4.6	GE Signa HDxt

BDI = Beck Depression Inventory; HCs = healthy controls; HDRS-17 = 17-item Hamilton Depression Rating Scale; m = months; MADRS = Montgomery and Åsberg Depression Rating Scale; MAOI = monoamine oxidase inhibitors; MDD = major depressive disorder; MRI = magnetic resonance imaging; NaSSA = noradrenergic/specific serotonergic agents; NDRI = norepinephrine dopamine reuptake inhibitor; pre-tx = pretreatment; post-tx = posttreatment; SSRI = specific serotonin reuptake inhibitors; SNRI = serotonin norepinephrine reuptake inhibitors; TCA = tricyclic antidepressants; w = weeks; y = years. Antidepressants include SSRI, SNRI TCA, NDRI, NaSSA, and MAOI.

**Table 2:** Sample characteristics and MRI procedures of included longitudinal studies.

Study (publication year)	Number (female)	Mean age (y)	Age of onset	Follow-up period	Illness duration (months)	No. of episodes	Mean depression severity		Therapy	Comorbidity with anxiety (rate%)	MRI procedures		
							Pretreat	Posttreat			Field strength	Voxel size (mm^3^)	MRI scanner
Arnone et al. (2013) [23]	23 (20)	36.3	22.0	8 weeks	NA	3.3	27.2 (MADRS)	3.9 (MADRS)	Citalopram	0/23 (0%)	1.5 T	0.875∗0.875∗1	Philips Intera
Cano et al. (2017) [31]	12 (6)	59.17	40.58	5 weeks	61.75 weeks	4.08	31.25 (HDRS-17)	2.92 (HDRS-17)	MECT+antidepressant+antipsychotics	3/12 (25%)	NA	27.9 (3.8)∗13.4 (1.7)∗10.4 (1.6)	Philips Achieva 3.0
Fang et al. (2015) [24]	20 (8)	59.2	56.1	8 weeks	3.6∗12 m	2.1	26.6 (HDRS-17)	6.4 (HDRS-17)	Antidepressants	NA	1.5 T	1.0∗1.0∗1.0	Siemens
Kong et al. (2014) [25]	24 (14)	36.12	NA	8 weeks	4.12	NA	21.64	3.4 (HDRS)	Fluoxetine	NA	1.5 T	1.5∗1.5∗1.5	GE Signa
Lemke et al. (2022) [26]	42 (24)	38.79	NA	2 year∗52 weeks	14.06	NA	4.36 (HDRS-17)	2.55 (HDRS-17)	Antidepressants	NA	3.0 T	NA	NA
Wang et al. (2017) [30]	23 (12)	38.74	33.90	≤3 weeks	70.35	NA	22.22 (HDRS-17)	3.83 (HDRS-17)	MECT+antidepressant+antipsychotics	NA	3.0 T	3.4∗3.4∗4.6	Signa HDxt, GE Healthcare, Buckinghamshire
Zaremba et al. (2018) [32]	23 (13)	32.5	NA	2.3∗52 weeks	15.3 weeks	2.7	22.4 (HDRS-17)	4.3 (HDRS-17)	Antidepressant+antipsychotic+mood stabilizers	NA	3.0 T	0.5∗0.5∗0.5	Philips Medical Systems

BDI = Beck Depression Inventory; HCs = healthy controls; HDRS-17 = 17-item Hamilton Depression Rating Scale; m = months; MADRS = Montgomery and Åsberg Depression Rating Scale; MRI = magnetic resonance imaging.

**Table 3:** Regional differences in GMV in patients with remitted MDD in meta-analysis of cross-sectional and longitudinal studies.

Brain regions	Maximum			Clusters	
	MNI coordinates (*x*, *y*, *z*)	SDM value	*p* value	No. of voxels	Breakdown (no. of voxels)
Remitted MDD > HCs					
Right superior temporal gyrus, BA 22	62, -24, 4	1.324	<0.001	693	Right superior temporal gyrus, BA 21, 22, 42, 48 (500)
					Right middle temporal gyrus, BA 21, 42 (193)
Right superior temporal gyrus, BA 48	50, -2, -6	1.087	0.003	119	Right temporal pole, superior temporal gyrus, BA 21, 22, 38, 48 (119)
Left anterior cingulate/paracingulate gyri, BA 25	4, 32, 8	1.309	<0.001	511	Left anterior cingulate/paracingulate gyri, BA 24, 25 (279)
					Right anterior cingulate/paracingulate gyri, BA 24, 32, 11 (163)
					Right median cingulate/paracingulate gyri, BA 24, 32 (127)
					Left median cingulate/paracingulate gyri, BA 24 (52)
Right striatum	10, 18, -8	1.212	0.001	99	Right striatum (94)
Right superior frontal gyrus, orbital part, BA 11	18, 62, -6	1.082	0.002	73	Right superior frontal gyrus, orbital part, BA 11 (73)
Remitted MDD < HCs					
Left amygdala, BA 28	-20, 0, -24	-1.189	<0.001	365	Left parahippocampal gyrus, BA 28, 34, 35, 36 (201)
					Left amygdala, BA 28, 34, 36 (87)
					Left hippocampus, BA 28, 35 (77)
Left inferior parietal (excluding supramarginal and angular) gyri, BA 39	-38, -58, 50	-1.331	<0.001	212	Left inferior parietal (excluding supramarginal and angular) gyri, BA 7, 39, 40 (212)
Left middle temporal gyrus, BA 37	-58, -58, 12	-1.330	<0.001	236	Left middle temporal gyrus, BA 21, 37, 39 (236)
Left insula, BA 48	-32, 20, 12	-1.280	<0.001	241	Left insula, BA 45, 47, 48 (123)
					Left inferior frontal gyrus, triangular part, BA 45, 47, 48 (97)
Right superior parietal gyrus, BA 7	30, -70, 56	-1.331	<0.001	139	Right superior parietal gyrus, BA 7 (139)
Remitted MDD follow‐up > baseline					
Left lenticular nucleus, putamen, BA 48	-24, 2, -8	2.472	0.001	803	Left striatum (190)
					Left lenticular nucleus, putamen, BA 48, BA 11, BA 25, BA 34 (188)
					Left amygdala, BA 34, BA 28, BA 36, BA 20, BA 48 (195)
					Left parahippocampal gyrus, BA 28, BA 36, BA 34, BA 35 (133)
					Left hippocampus (97)
Left median cingulate/paracingulate gyri	-2, -26, 48	2.201	<0.001	583	Left median cingulate/paracingulate gyri. BA 23 (257)
					Right median cingulate/paracingulate gyri (225)
					Left paracentral lobule, BA 4 (101)
Left precuneus	-4, -64, 48	1.933	0.003	36	Left precuneus, BA 7 (36)
Remitted MDD follow‐up < baseline					
Left gyrus rectus	-2, 48, -22	-1.666	<0.001	849	Left gyrus rectus, BA 11 (416)
					Right gyrus rectus (208)
					Left superior frontal gyrus, medial orbital, BA 11, BA 10 (175)
					Left superior frontal gyrus, orbital part, BA 11 (50)

MDD = major depressive disorder.

## Data Availability

The original data is available from the corresponding author upon request.

## Supplementary Materials

Supplementary Materials See Tables S1–S4 and Figures S1–S4 in the Supplementary Material.
