# Supplementary material for: Meta-Analysis of Brain Volumetric Abnormalities in Patients with Remitted Major Depressive Disorder
Source: Depress Anxiety. 2024 May 15;2024:6633510. doi: 10.1155/2024/6633510 (PMC11919220; doi:10.1155/2024/6633510)
Supplement: Supplementary Materials — See Tables S1–S4 and Figures S1–S4 in the Supplementary Material. [file 6633510.f1.docx]

**Supplementary Materials**

Table S1. Subgroup analysis of GMV in patients with remitted MDD after pharmacological treatment in comparison with HCs.

Table S2. The jackknife analysis in GMV in patients with remitted MDD in comparison with HCs.

Table S3. The jackknife analysis in GMV in patients with remitted MDD after antidepressant medication in comparison with HCs in subgroup analysis.

Table S4. The jackknife analysis in GMV in patients with remitted MDD from baseline to follow-up.

Figure S1. Subgroup analysis of GMV in patients with remitted MDD after pharmacological treatment in comparison with HCs.

Figure S2. Results of funnel plot analysis for the pooled analysis of cross-section datasets in remitted MDD patients in comparison with HCs.

Figure S3. Results of funnel plot analysis for the subgroup analysis of cross-section datasets.

Figure S4. Results of funnel plot analysis for the analysis of longitudinal datasets in patients with remitted MDD.

References

**Table S1 Subgroup analysis of GMV in patients with remitted MDD after pharmacological treatment in comparison with HCs.**

| **Brain Regions** | **Maximum** | | |  | **Clusters** | |
| --- | --- | --- | --- | --- | --- | --- |
|  | **MNI coordinates,**  **x,y,z** | **SDM value** | **P-value** |  | **No. of voxels** | **Breakdown (No. of voxels)** |
| **remitted MDD>HCs** | |  |  |  |  |  |
| **Left anterior cingulate / paracingulate gyri, BA 25** | 0,34,6 | 1.205 | <0.001 |  | 265 | Left anterior cingulate / paracingulate gyri, BA 24, 25 (136) |
|  |  |  |  |  |  | Right anterior cingulate / paracingulate gyri, BA 24, 32, 11 (75) |
|  |  |  |  |  |  | Corpus callosum (54) |
| **Right gyrus rectus, BA 11** | 12,20,-14 | 1.199 | <0.001 |  | 179 | Right striatum (72) |
|  |  |  |  |  |  | Right gyrus rectus, BA 11, 25 (56) |
|  |  |  |  |  |  | Corpus callosum (51) |
| **Left inferior frontal gyrus, triangular part, BA 48** | -36,14,26 | 1.001 | 0.001 |  | 62 | Left inferior frontal gyrus, triangular part, opercular part, BA 44,48 (62) |
| **remitted MDD< HCs** |  |  |  |  |  |  |
| **Left inferior parietal (excluding supramarginal and angular) gyri, BA 39** | -40,-58,48 | -1.315 | <0.001 |  | 294 | Left inferior parietal (excluding supramarginal and angular) gyri, BA 7,39,40 (294) |
| **Left middle temporal gyrus, BA 37** | -56,-62,14 | -1.318 | <0.001 |  | 235 | Left middle temporal gyrus, BA 21,31(235) |
| **Right superior parietal gyrus, BA 7** | 28,-64,58 | -1.313 | <0.001 |  | 213 | Right superior parietal gyrus, BA 7 (172) |
|  |  |  |  |  |  | Right angular gyrus, BA 7 (41) |
| **Left insula, BA 48** | -32,20,12 | -1.354 | <0.001 |  | 172 | Left insula, BA 45, 47, 48 (121) |
|  |  |  |  |  |  | Left superior longitudinal fasciculus III (51) |

HCs, healthy controls; MDD, major depressive disorder.

Table S2 **The jackknife analysis in GMV in patients with remitted MDD in comparison with HCs.**

| Studies | Increased GMV | | | | | | |  | Decreased GMV | | | | | | |
| --- | --- | --- | --- | --- | --- | --- | --- | --- | --- | --- | --- | --- | --- | --- | --- |
|  | **R STG** | **R MTG** | **R TP** | **B ACG** | **B MCG** | **R STA** | **R SFG** |  | **L PHG** | **L AMYG** | **L HIP** | **L IPG** | **L INS** | **L IFG** | **R SPG** |
| Arnone et al. 2013 [1] | Yes | Yes | Yes | Yes | Yes | Yes | Yes |  | Yes | Yes | Yes | Yes | Yes | Yes | Yes |
| Fang et al. 2015 [2] | NO | NO | NO | Yes | Yes | Yes | Yes |  | NO | NO | NO | Yes | NO | NO | Yes |
| Klauser et al. 2015 [3] | Yes | Yes | Yes | Yes | Yes | Yes | Yes |  | Yes | Yes | Yes | Yes | Yes | Yes | Yes |
| Kong et al. 2014 [4] | Yes | Yes | Yes | Yes | Yes | Yes | NO |  | Yes | Yes | Yes | Yes | Yes | Yes | Yes |
| Lemke et al. 2022 [5] | Yes | Yes | Yes | Yes | Yes | Yes | Yes |  | Yes | Yes | Yes | NO | Yes | Yes | NO |
| Li et al. 2010 [6] | Yes | Yes | Yes | Yes | Yes | Yes | Yes |  | Yes | Yes | Yes | Yes | Yes | Yes | Yes |
| Liu et al. 2014 [7] | Yes | Yes | Yes | Yes | Yes | Yes | Yes |  | Yes | Yes | Yes | Yes | NO | NO | Yes |
| Salvadore et al. 2011 [8] | Yes | Yes | Yes | NO | NO | Yes | Yes |  | Yes | Yes | Yes | Yes | Yes | Yes | Yes |
| Serra-Blasco et al. 2013 [9] | Yes | Yes | Yes | Yes | Yes | Yes | Yes |  | Yes | Yes | Yes | Yes | Yes | Yes | Yes |
| Takamiya et al. 2021 [10] | NO | NO | NO | Yes | NO | Yes | NO |  | Yes | Yes | Yes | Yes | Yes | Yes | Yes |
| Wang et al. 2017 [11] | Yes | Yes | Yes | Yes | Yes | Yes | Yes |  | NO | NO | NO | Yes | Yes | Yes | Yes |

**Abbreviation:** ACC, anterior cingulate cortex; AMY, amygdala; GMV, grey matter volumes; HCs, healthy controls; IFG, inferior frontal gyrus; INS, insula; IPG, inferior parietal gyri; L left; MCC, median cingulate cortex; MDD, major depressive disorder; MTG, middle temporal gyrus; R, right; SPG, superior parietal gyrus; STG, superior temporal gyrus; STR, striatum.

**Table S3 The jackknife analysis in GMV in patients with remitted MDD after antidepressant medication in comparison with HC in subgroup analysis.**

|  | Increased GM volumes | | | | |  | Decreased GM volumes | | | |
| --- | --- | --- | --- | --- | --- | --- | --- | --- | --- | --- |
|  | **L ACG** | **R ACC** | **RGR** | **R STR** | **L IFG** |  | **L MTG** | **L IPG** | **L INS** | **R SPG** |
| Arnone et al. 2013 [1] | Yes | Yes | Yes | Yes | Yes |  | Yes | Yes | Yes | Yes |
| Fang et al. 2015 [2] | Yes | Yes | Yes | Yes | Yes |  | Yes | Yes | NO | Yes |
| Klauser et al. 2015 [3] | Yes | Yes | Yes | Yes | Yes |  | Yes | Yes | Yes | Yes |
| Kong et al. 2014 [4] | Yes | Yes | Yes | Yes | NO |  | Yes | Yes | Yes | Yes |
| Lemke et al. 2022 [5] | Yes | Yes | Yes | Yes | Yes |  | NO | NO | Yes | Yes |
| Li et al. 2010 [6] | Yes | Yes | Yes | Yes | Yes |  | Yes | Yes | Yes | Yes |
| Liu et al. 2014 [7] | Yes | Yes | Yes | Yes | Yes |  | Yes | Yes | Yes | Yes |
| Salvadore et al. 2011 [8] | NO | NO | NO | Yes | Yes |  | Yes | Yes | Yes | Yes |
| Serra-Blasco et al. 2013 [9] | Yes | Yes | Yes | Yes | Yes |  | Yes | Yes | Yes | Yes |

**Abbreviation:** ACC, anterior cingulate cortex; GMV, grey matter volumes; HCs, healthy controls; IFG, inferior frontal gyrus; INS, insula; IPG, inferior parietal gyri; L left; MDD, major depressive disorder; MTG, middle temporal gyrus; R, right; SPG, superior parietal gyrus.

**Table S4. The jackknife analysis in GMV in patients with remitted MDD from baseline to follow-up.**

| Studies | Increased GMV | | | | | | |  | | Decreased GMV | | | |
| --- | --- | --- | --- | --- | --- | --- | --- | --- | --- | --- | --- | --- | --- |
|  | **L STR** | **L AMY** | **L PHP** | **L HP** | **B MCG** | **L PREC** |  | | **L GR** | | **L SFG** | **R GR** |  |
| Arnone et al. 2013 [1] | Yes | Yes | Yes | Yes | Yes | Yes |  | | Yes | | Yes | Yes |  |
| Cano et al. 2017 [12] | NO | NO | NO | NO | Yes | Yes |  | | Yes | | Yes | Yes |  |
| Fang et al. 2015 [2] | Yes | Yes | Yes | Yes | NO | NO |  | | NO | | NO | NO |  |
| Kong et al. 2014 [4] | Yes | Yes | Yes | Yes | Yes | Yes |  | | Yes | | Yes | Yes |  |
| Lemke et al. 2022 [5] | Yes | Yes | Yes | Yes | Yes | Yes |  | | Yes | | Yes | Yes |  |
| Wang et al. 2017 [11] | Yes | NO | NO | NO | Yes | Yes |  | | Yes | | Yes | Yes |  |
| Zaremba et al. 2018 [13] | Yes | Yes | Yes | Yes | Yes | Yes |  | | Yes | | Yes | Yes |  |

**Abbreviation:** AMY, amygdala; GMV, grey matter volumes; GR, gyrus rectus; HP, hippocampus; L left; MCC, median cingulate cortex; MDD, major depressive disorder; PHP, parahippocampal gyrus; PREC, precuneus; PUT, putamen; R, right; STR, striatum.

**Figure S1 Subgroup analysis of GMV in patients with remitted MDD after pharmacological treatment in comparison with HCs.**


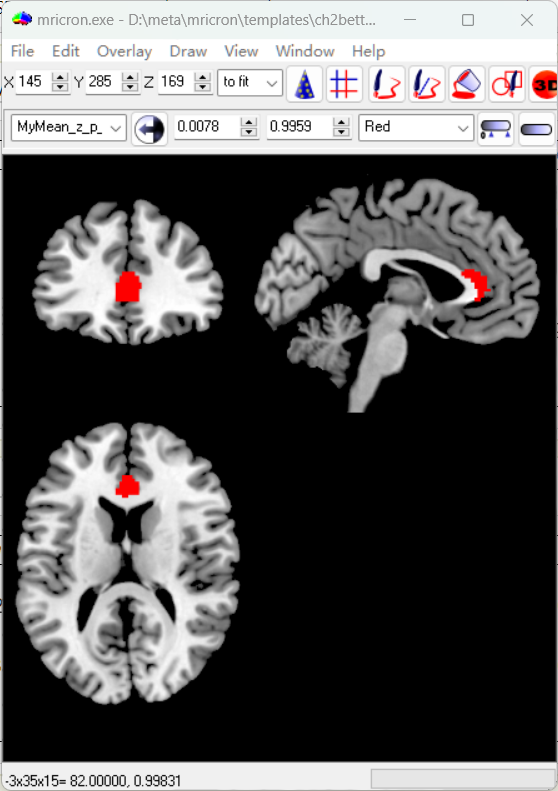


**B ACC**


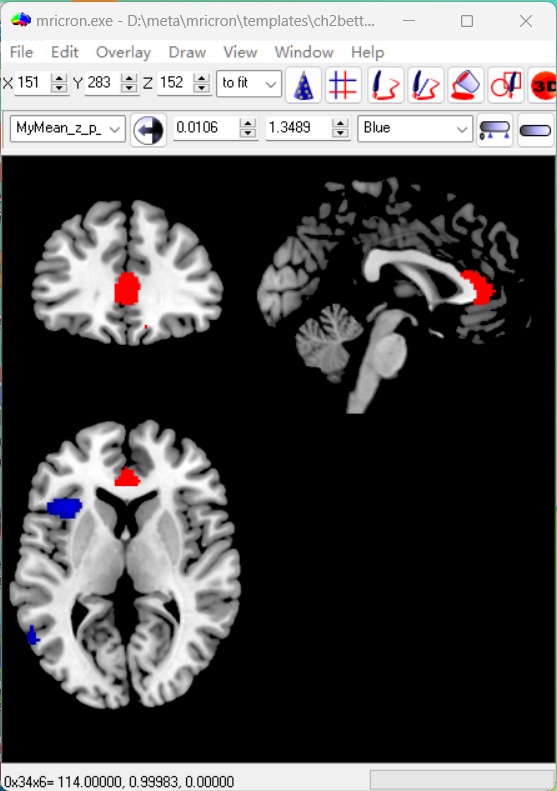

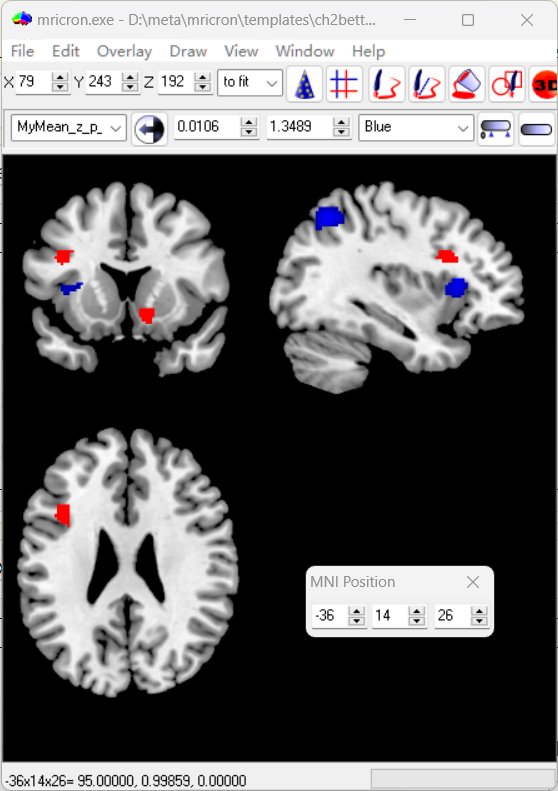

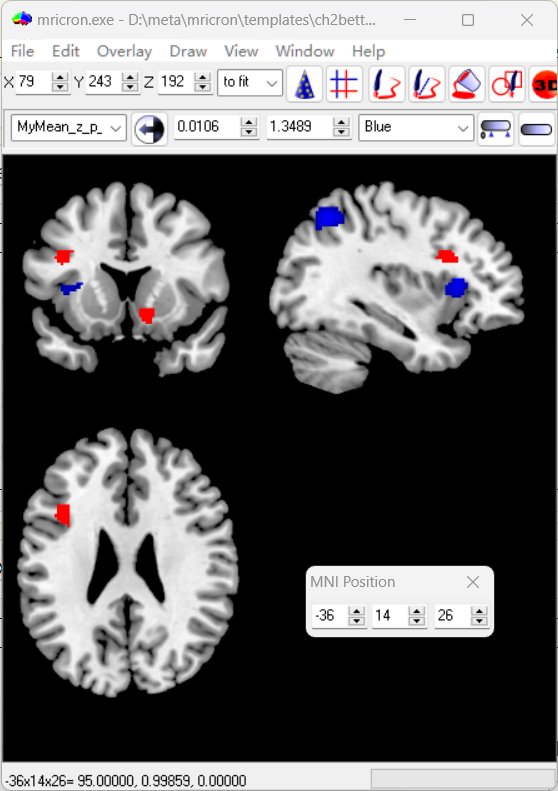


**L IFG**


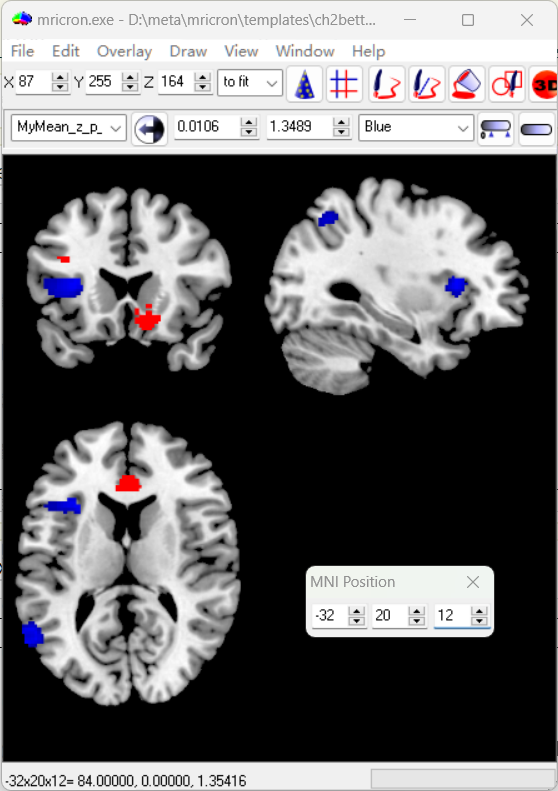

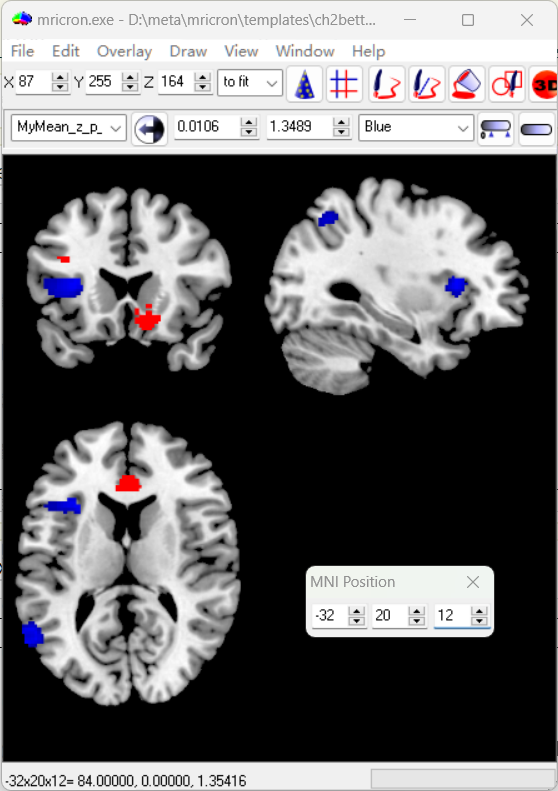

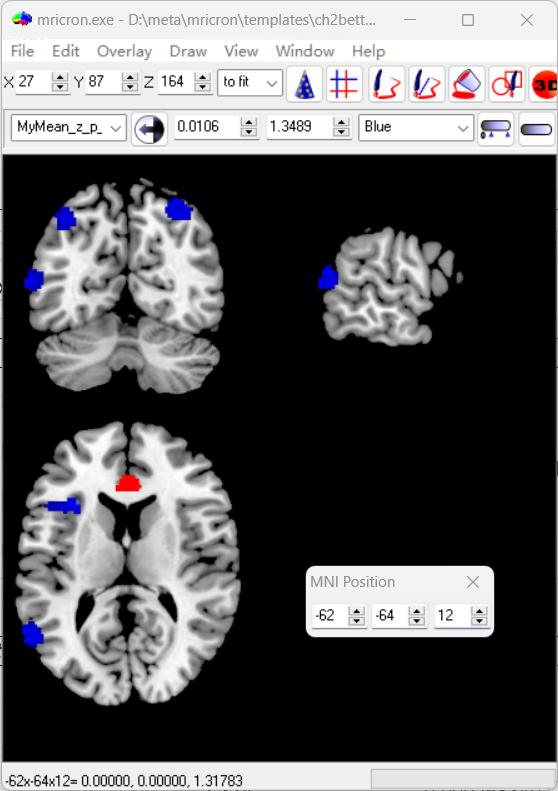


**L INS**


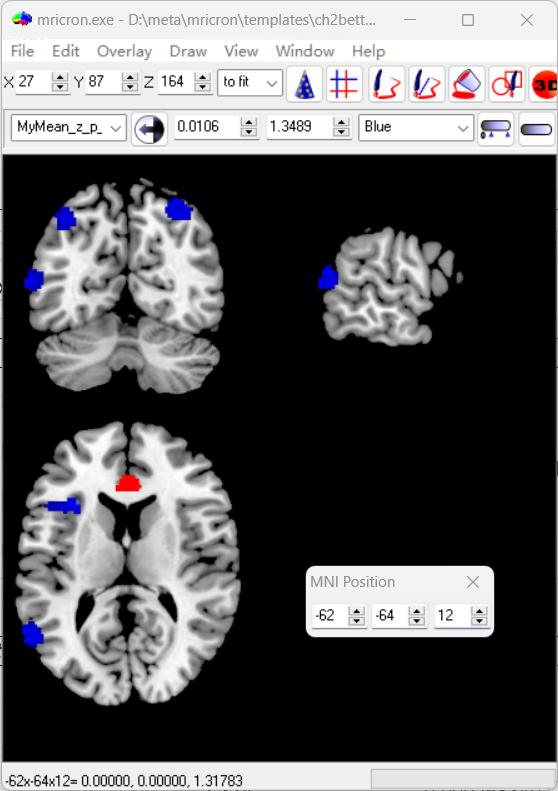

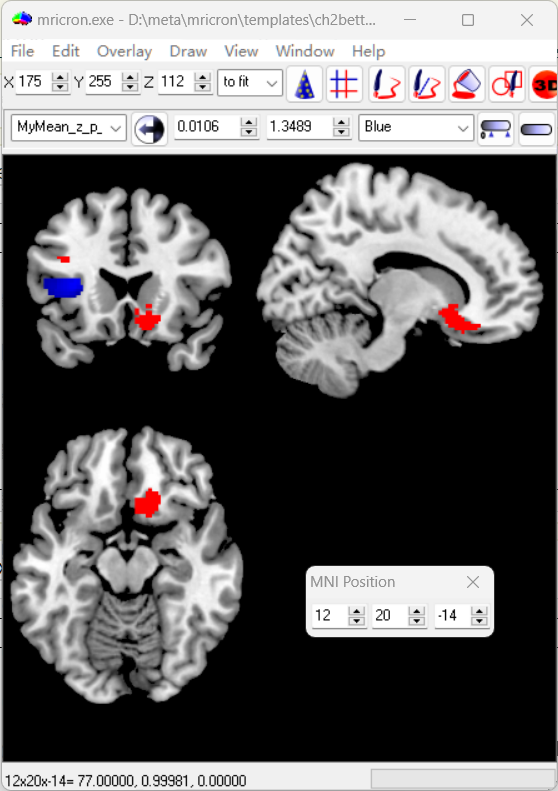

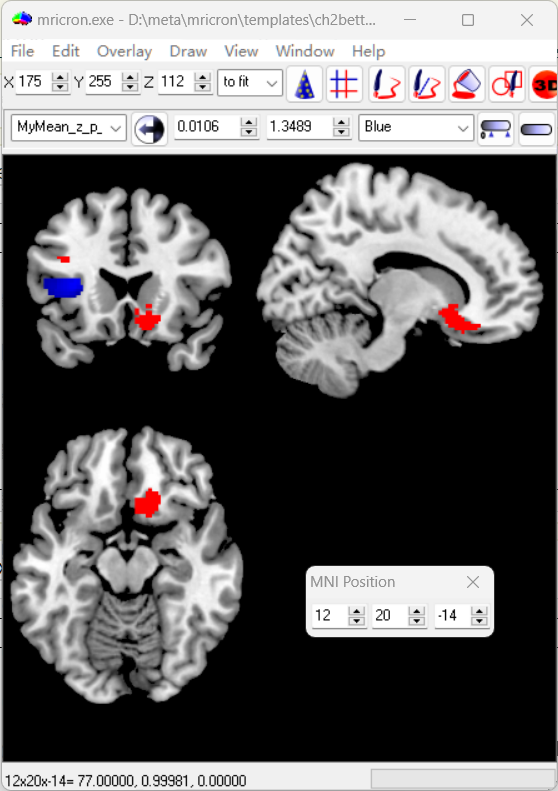


**R STR**


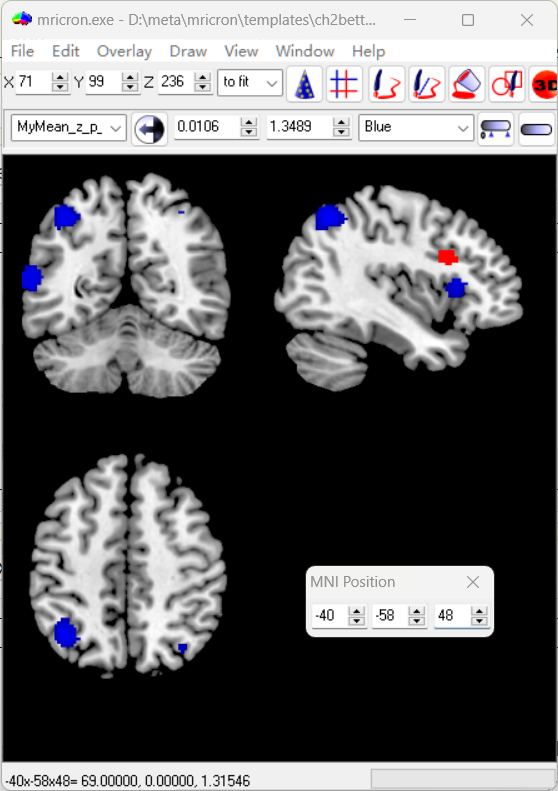

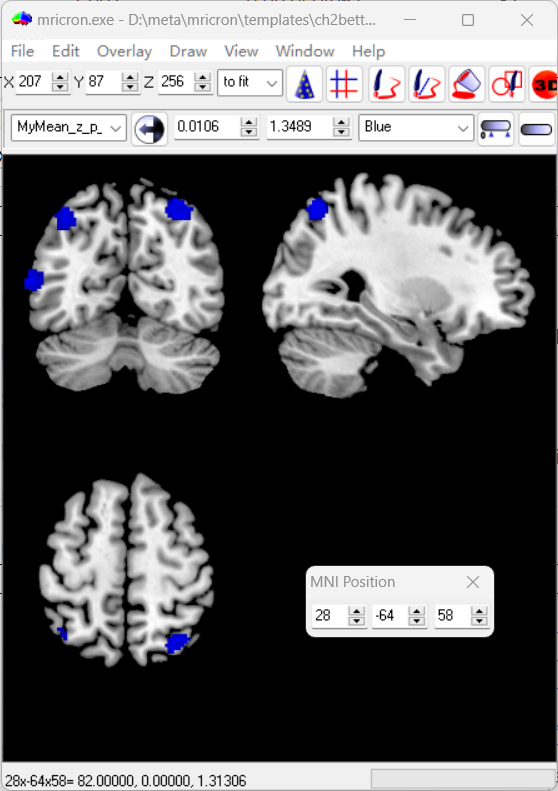


**L MTG**

**R SPG**

**L IPG**

**R STR**

**R GR**

**Abbreviation:** ACC, anterior cingulate cortex; GMV, grey matter volumes; HC, healthy controls; IFG, inferior frontal gyrus; INS, insula; IPG, inferior parietal gyri; L left; MDD, major depressive disorder; MTG, middle temporal gyrus; R, right; SPG, superior parietal gyrus.

**Figure S2.** **Results of funnel plot analysis for the pooled analysis of cross-section datasets in remitted MDD patients in comparison with HCs.**


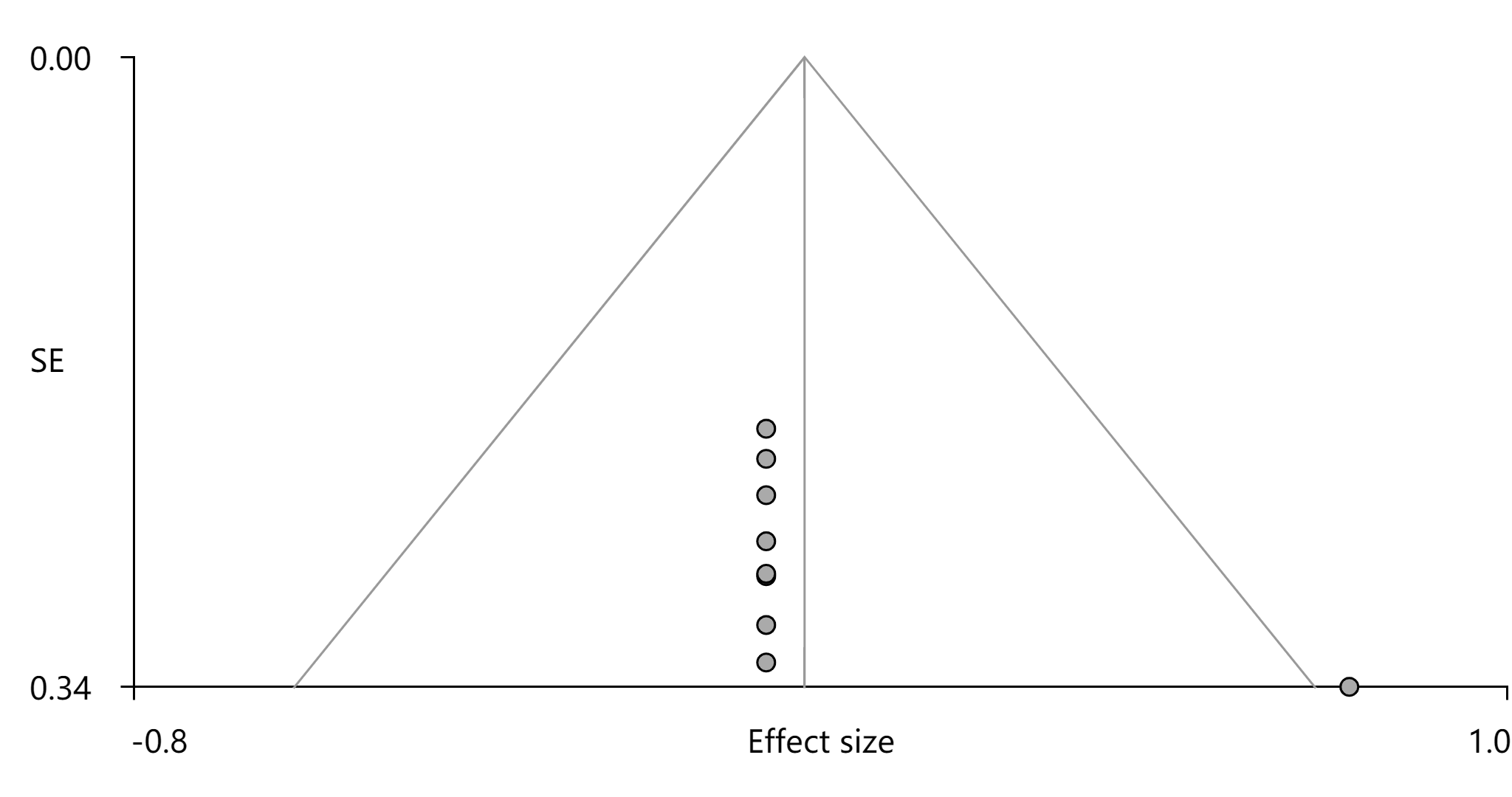

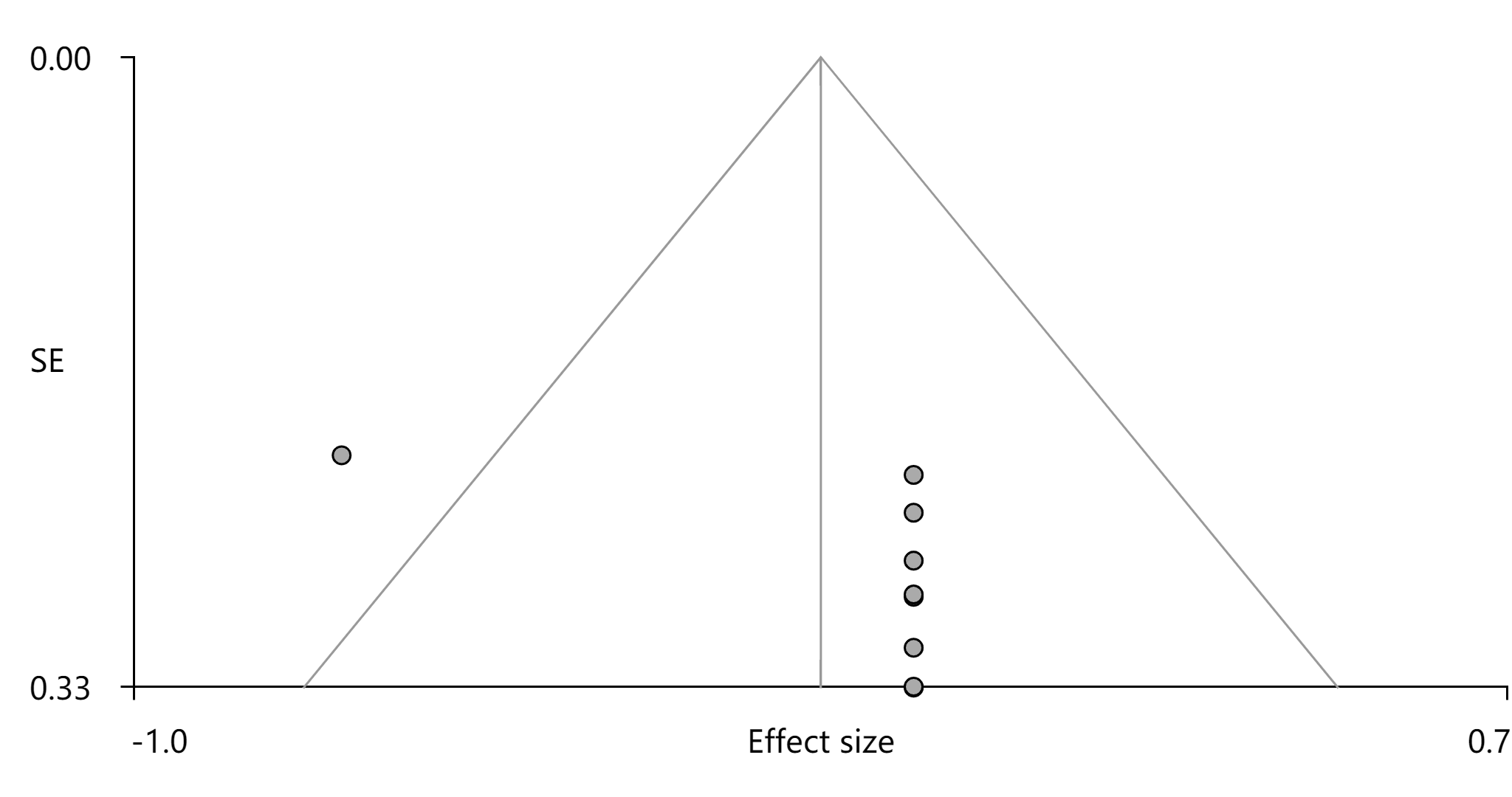


(2) R STG（BA48）：Bias: 2.08, t: 1.50, df: 7, p: 0.177

(1) R STG（BA22）：Bias: 2.08, t: 1.50, df: 7, p: 0.177


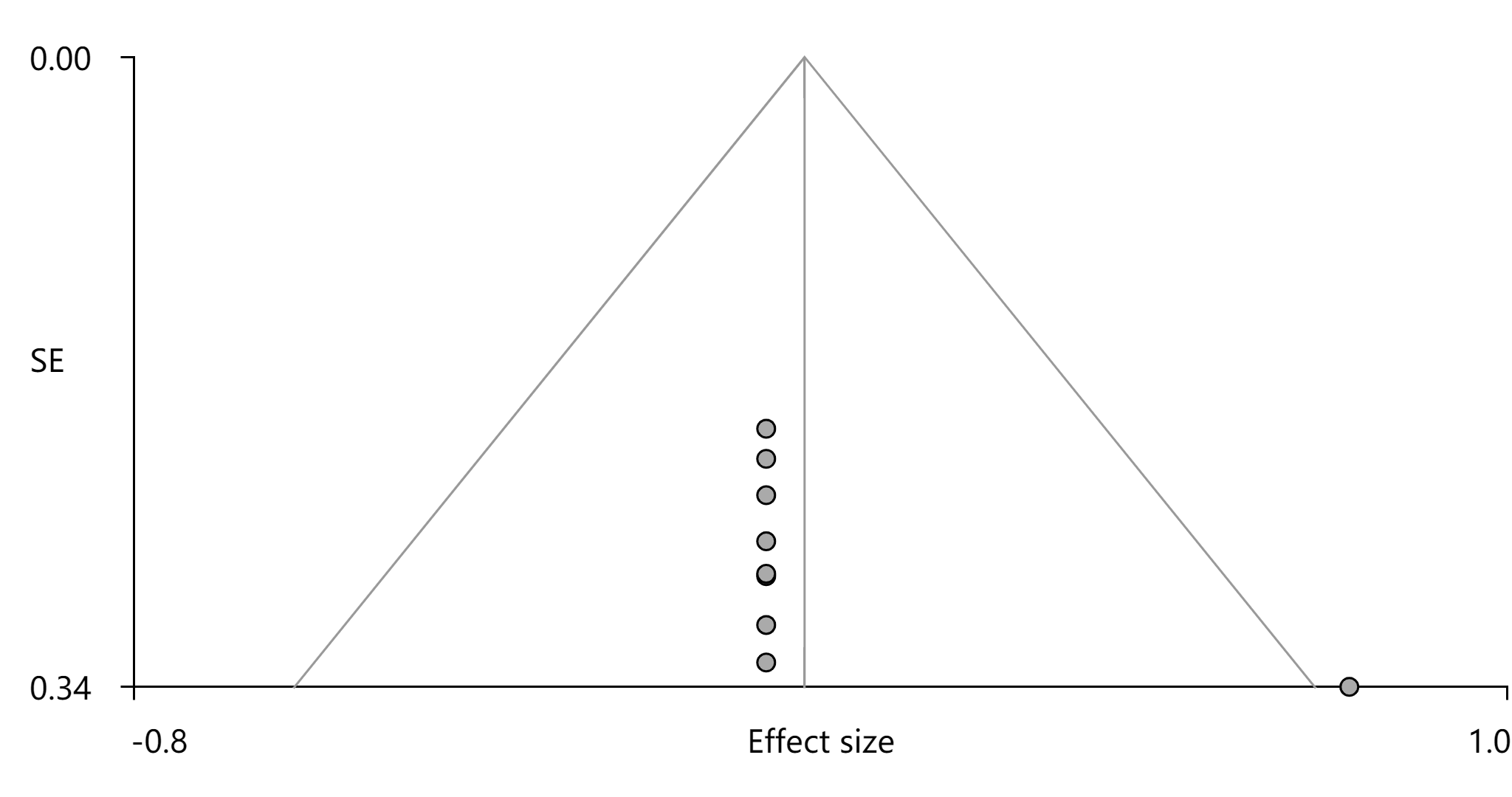


(6) L AMY：Bias: -1.91, t: -1.48, df: 7, p: 0.183


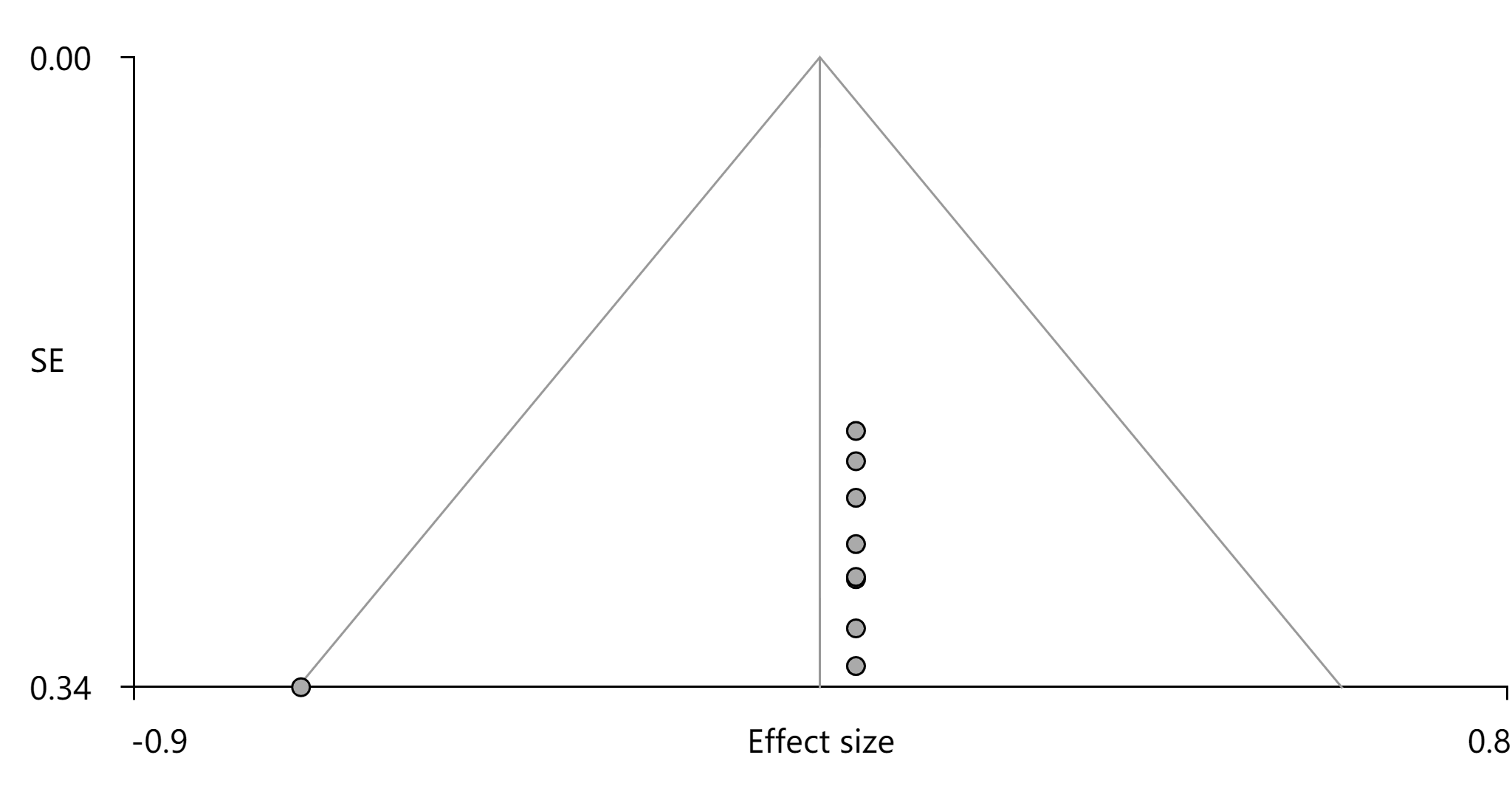


(7) L IPG: Bias: 3.64, t: 1.81, df: 7, p: 0.114

(3) L ACC：Bias: -2.17, t: -0.99, df: 7, p: 0.354


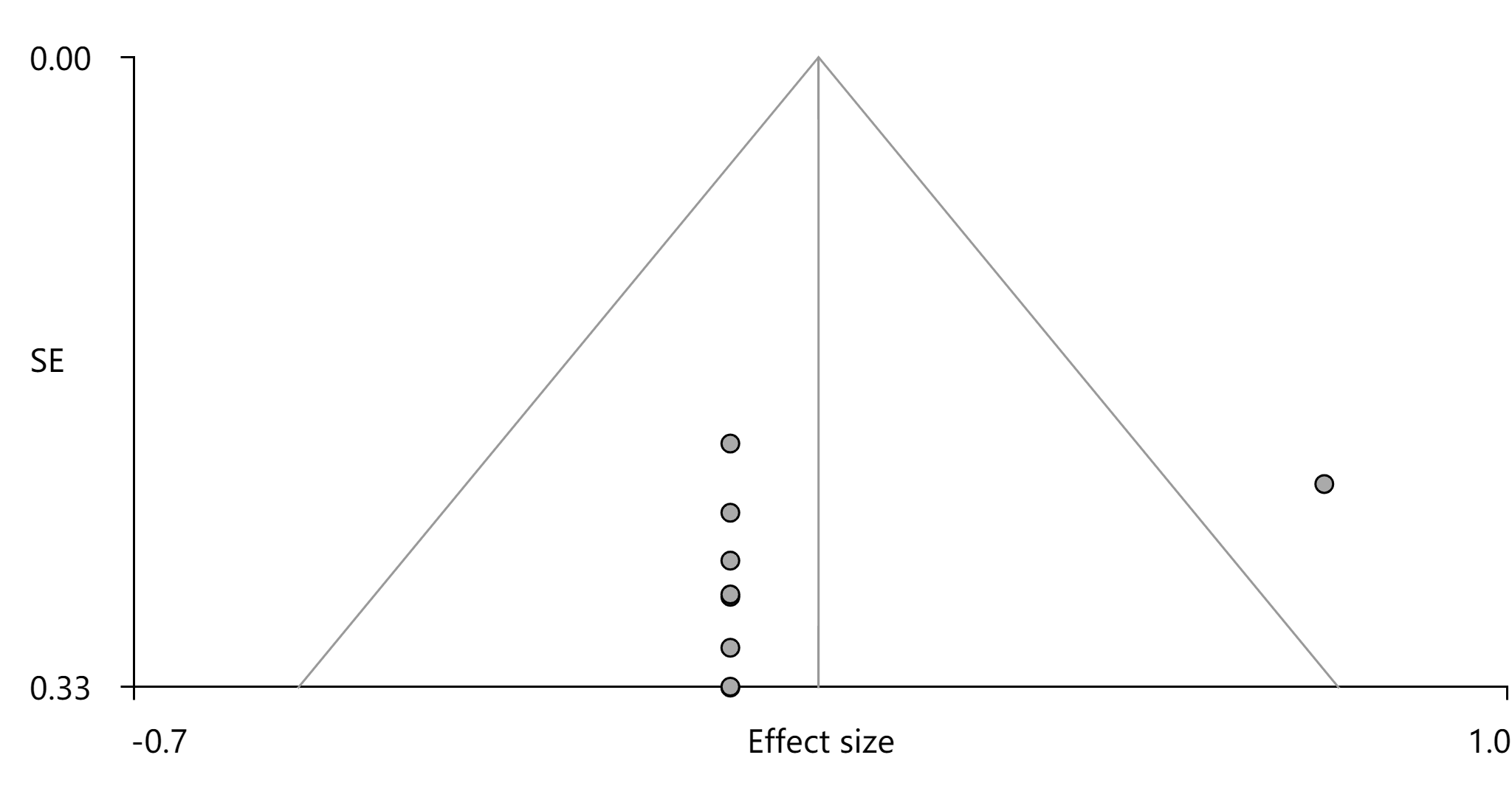


(8) L MTG:Bias: 3.64, t: 1.81, df: 7, p: 0.114


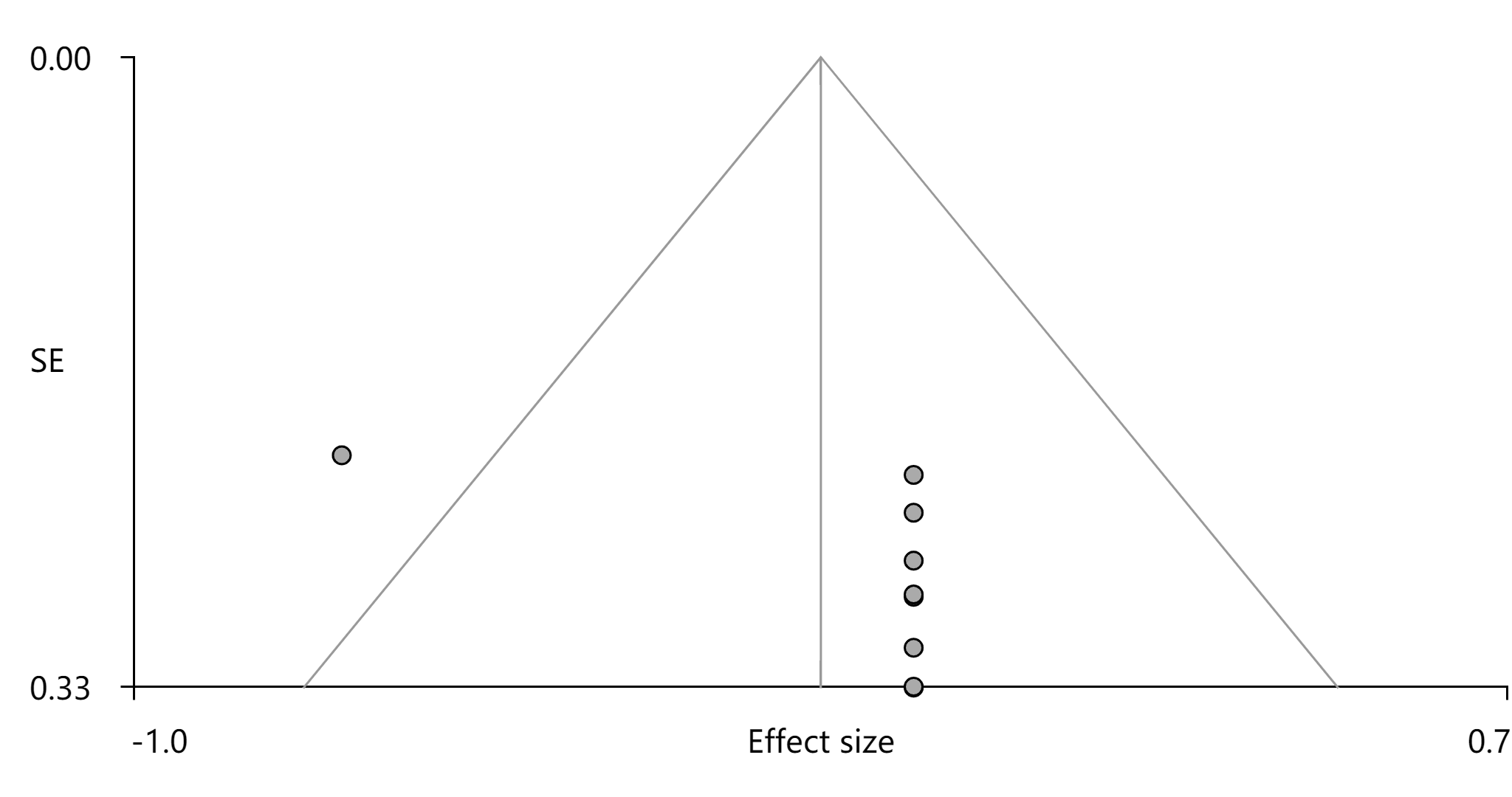


(4) R STR ：Bias: -2.17, t: -0.99, df: 7, p: 0.354


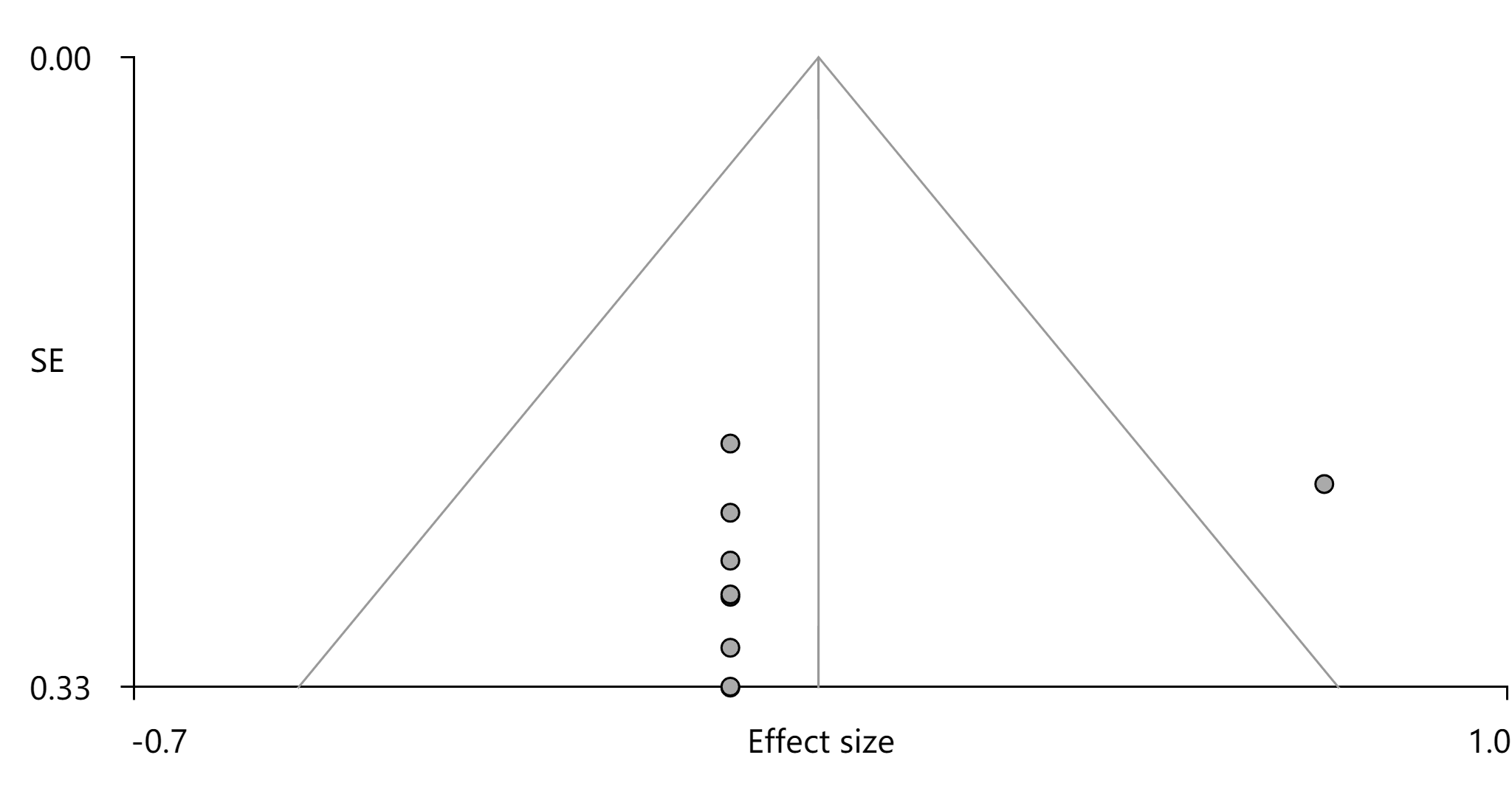


(9) L INS:Bias: -5.08, t: -2.79, df: 7, p: 0.027


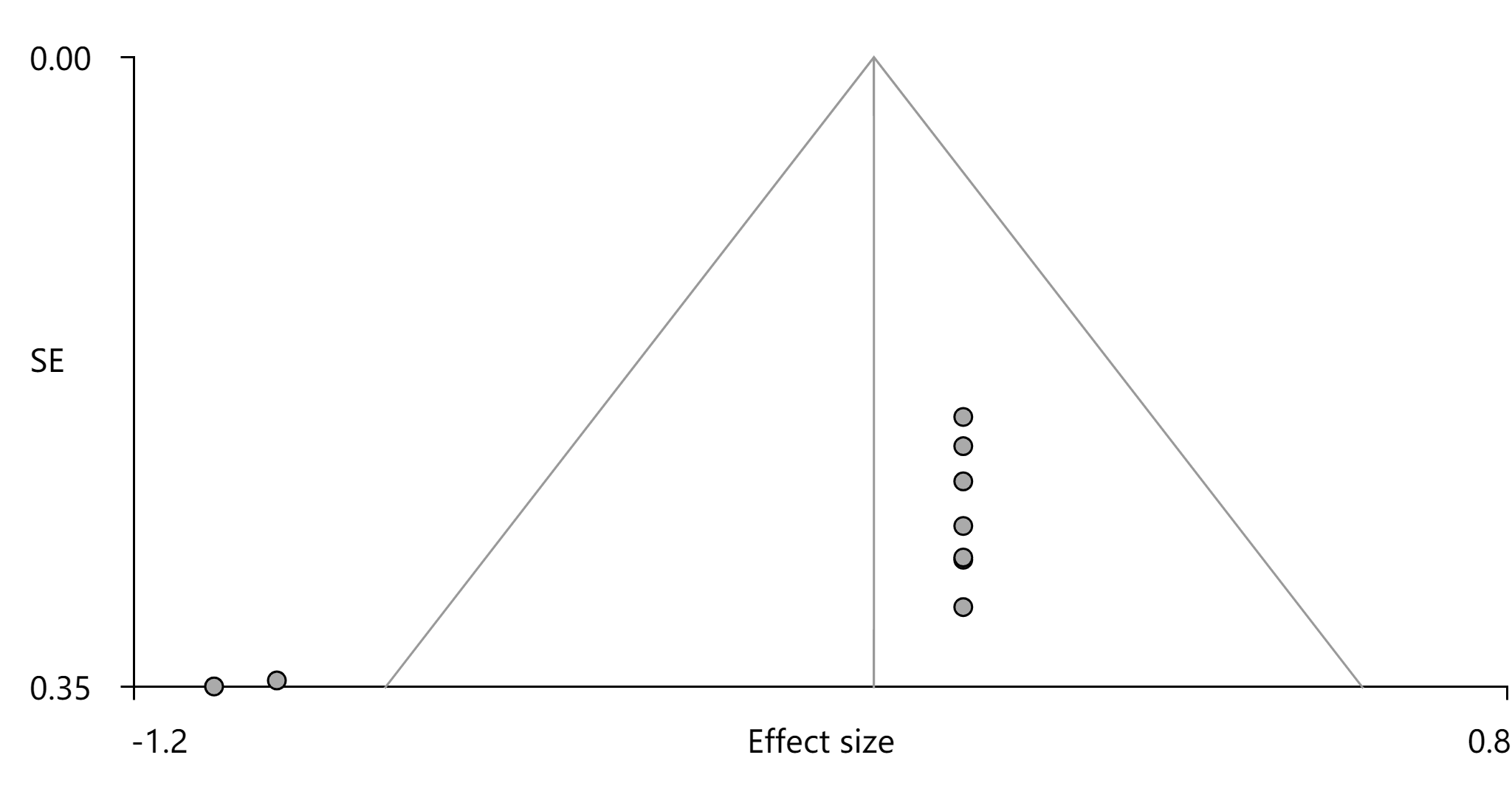


(10) R SPG: Bias: 3.64, t: 1.81, df: 7, p: 0.114

(5) R SFG：Bias: 1.69, t: 0.76, df: 7, p: 0.473


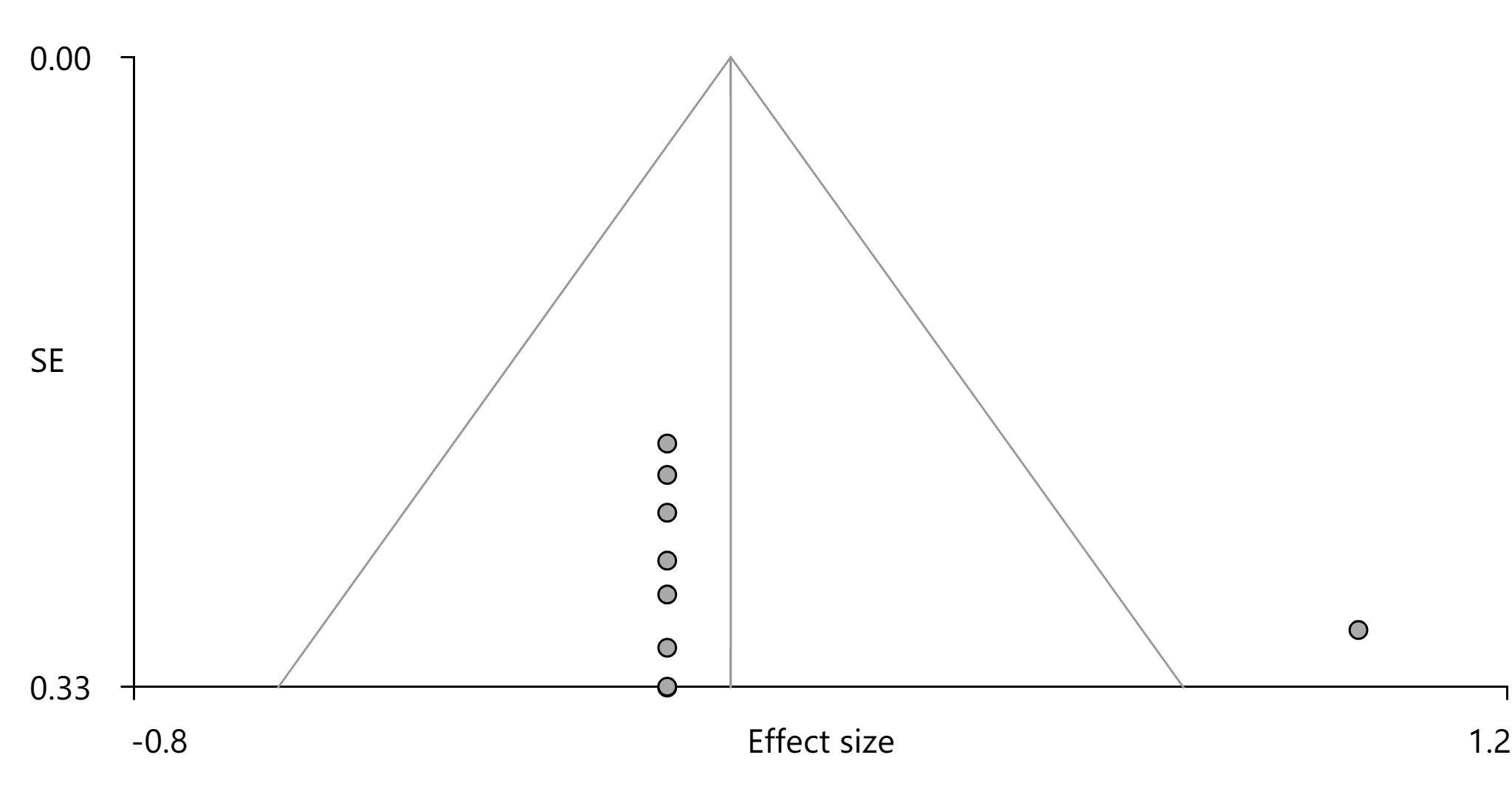

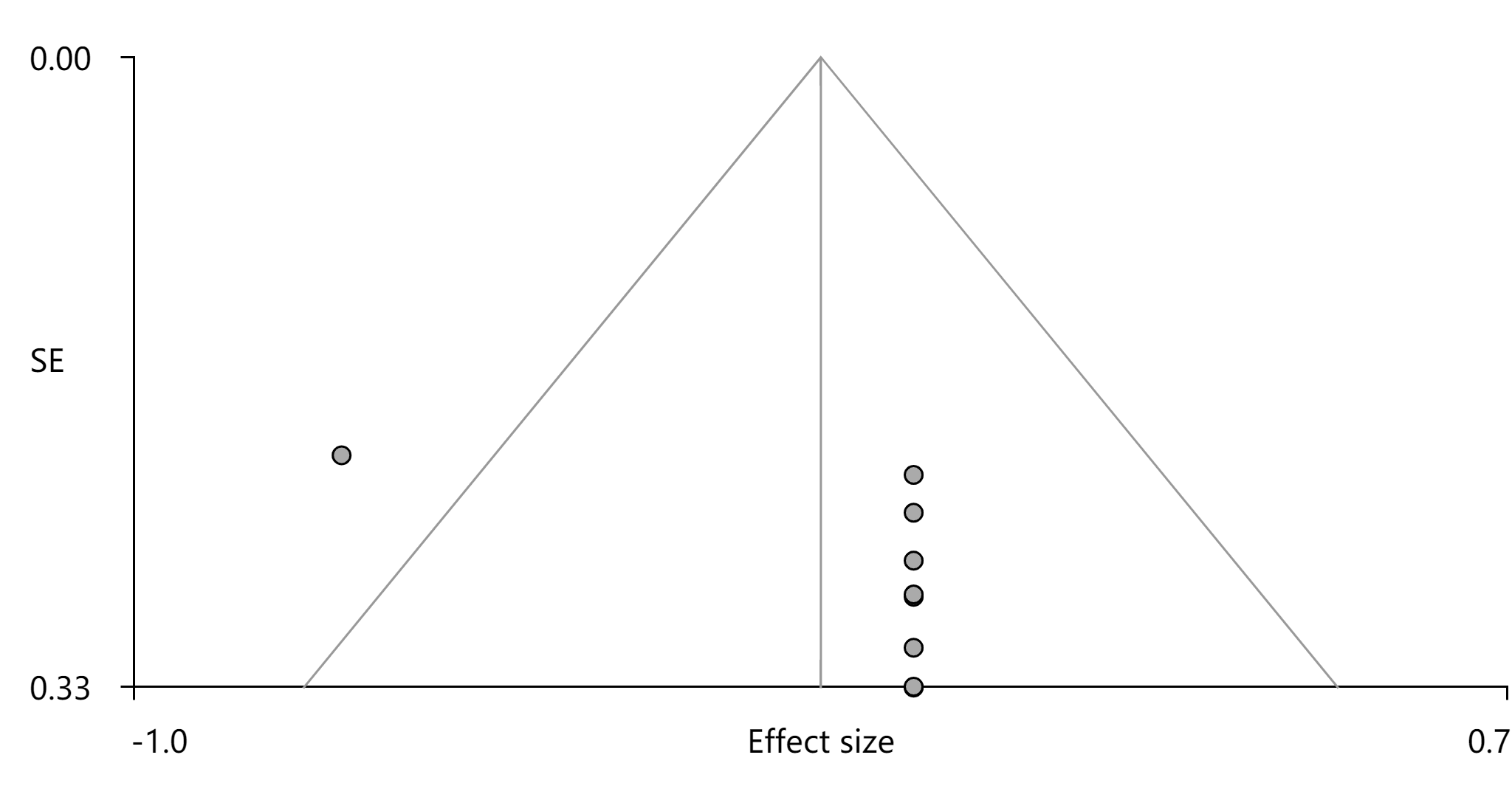


**Abbreviation:** ACC, anterior cingulate cortex; AMY, amygdala; IFG, inferior frontal gyrus; INS, insula; IPG, inferior parietal gyri; L left; MCC, median cingulate cortex; MTG, middle temporal gyrus; R, right; SPG, superior parietal gyrus; STG, superior temporal gyrus; STR, striatum.

**Figure S3 Results of funnel plot analysis for the subgroup analysis of cross-section datasets.**


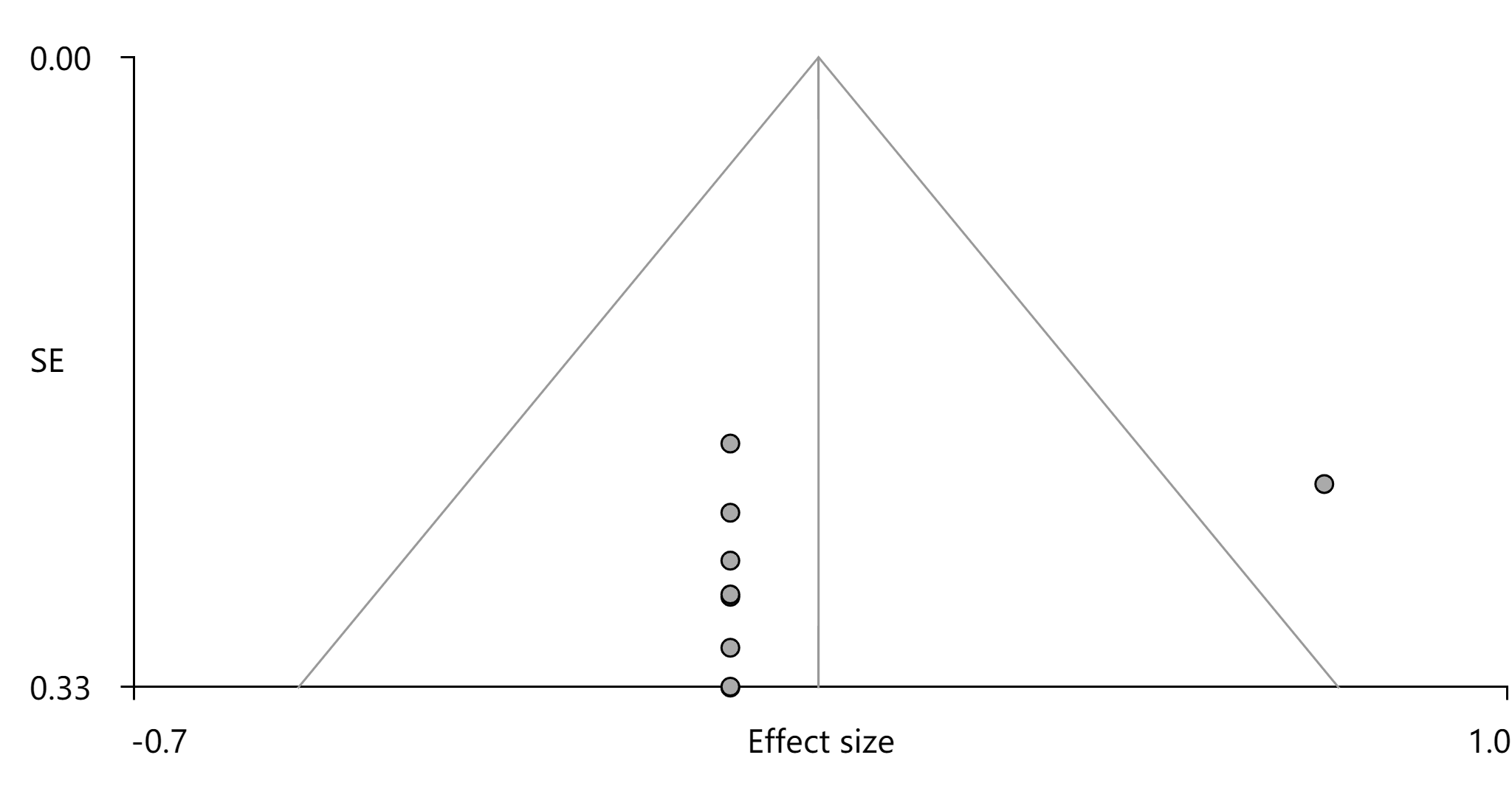

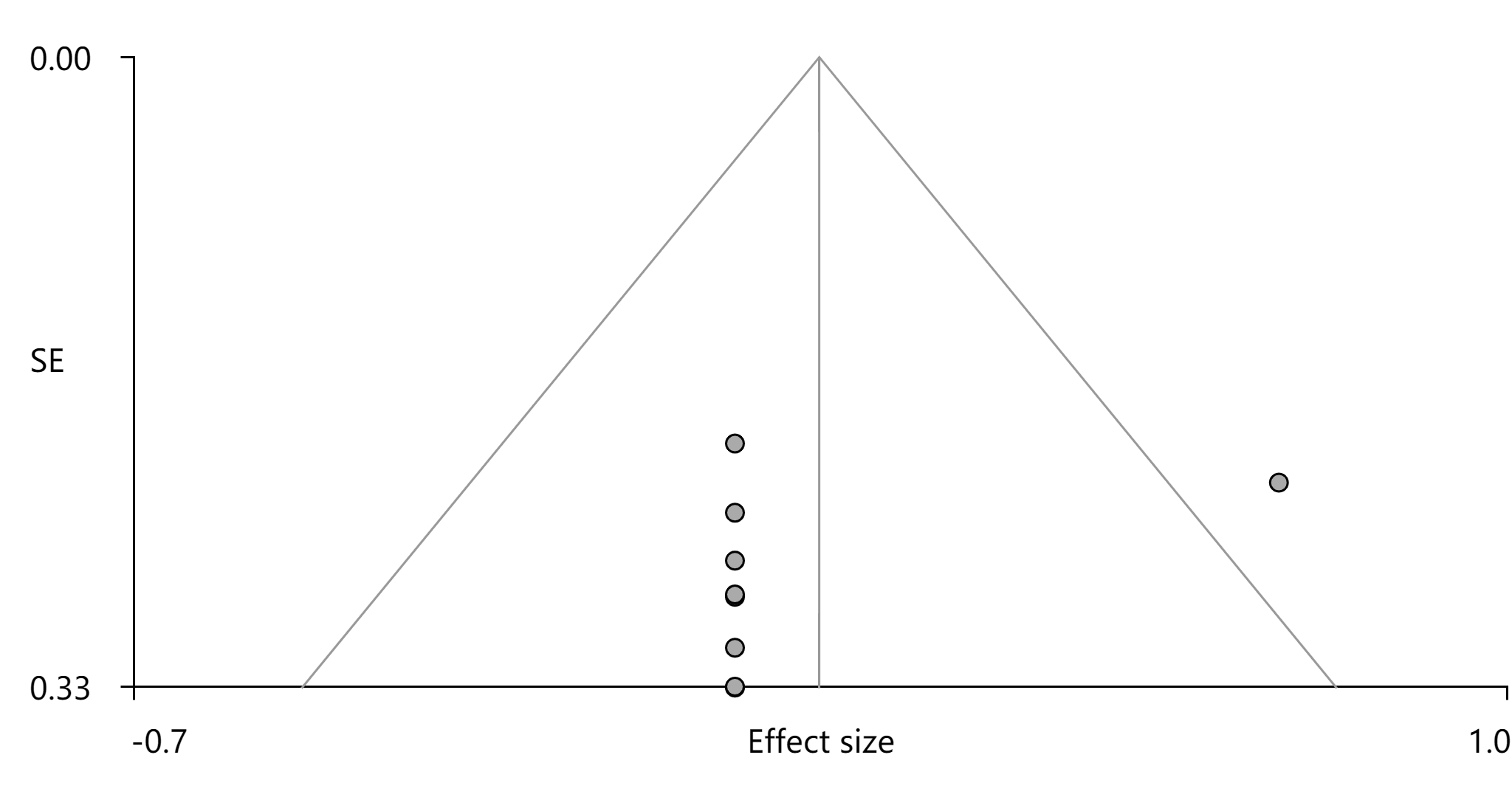

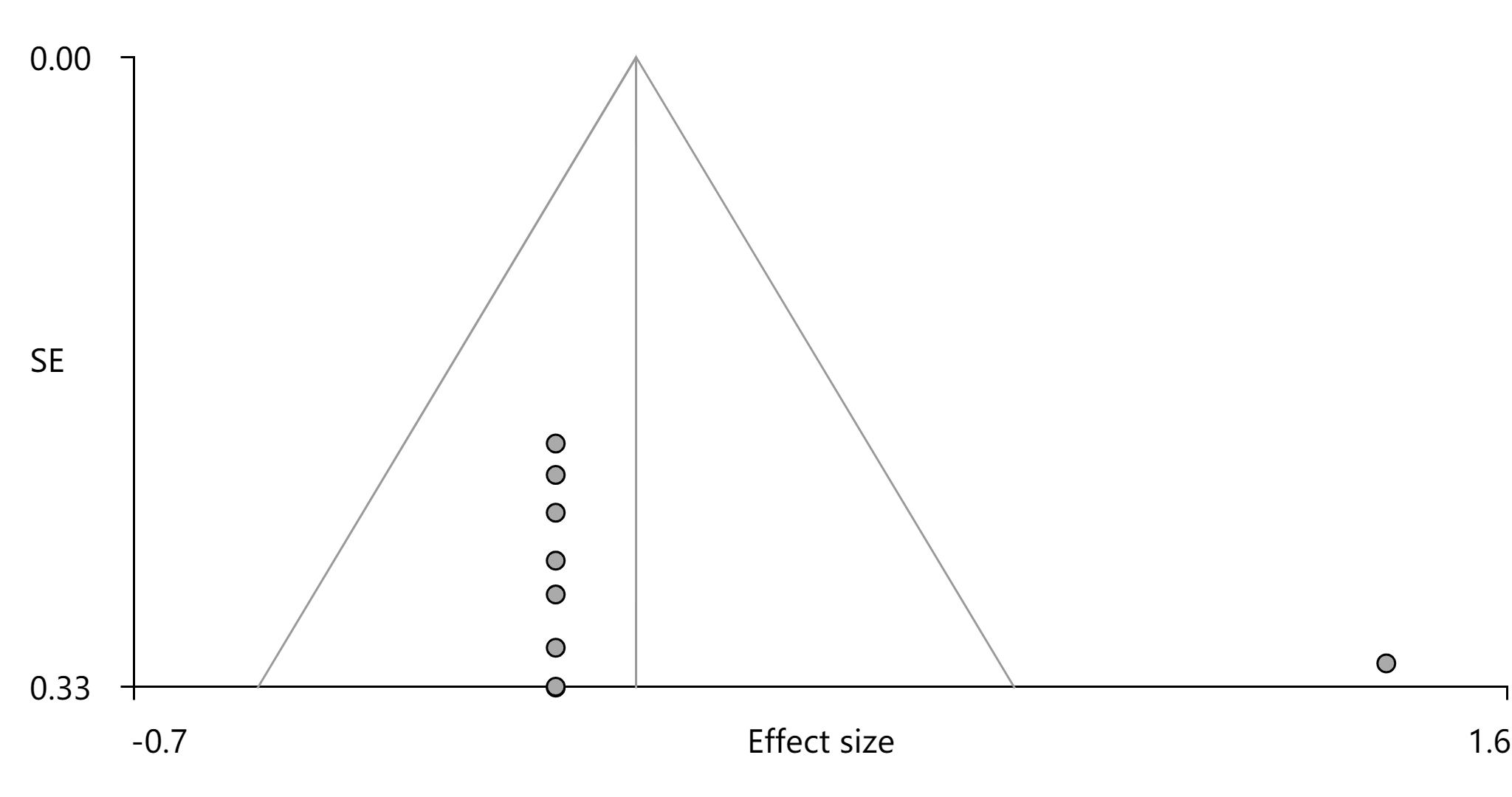

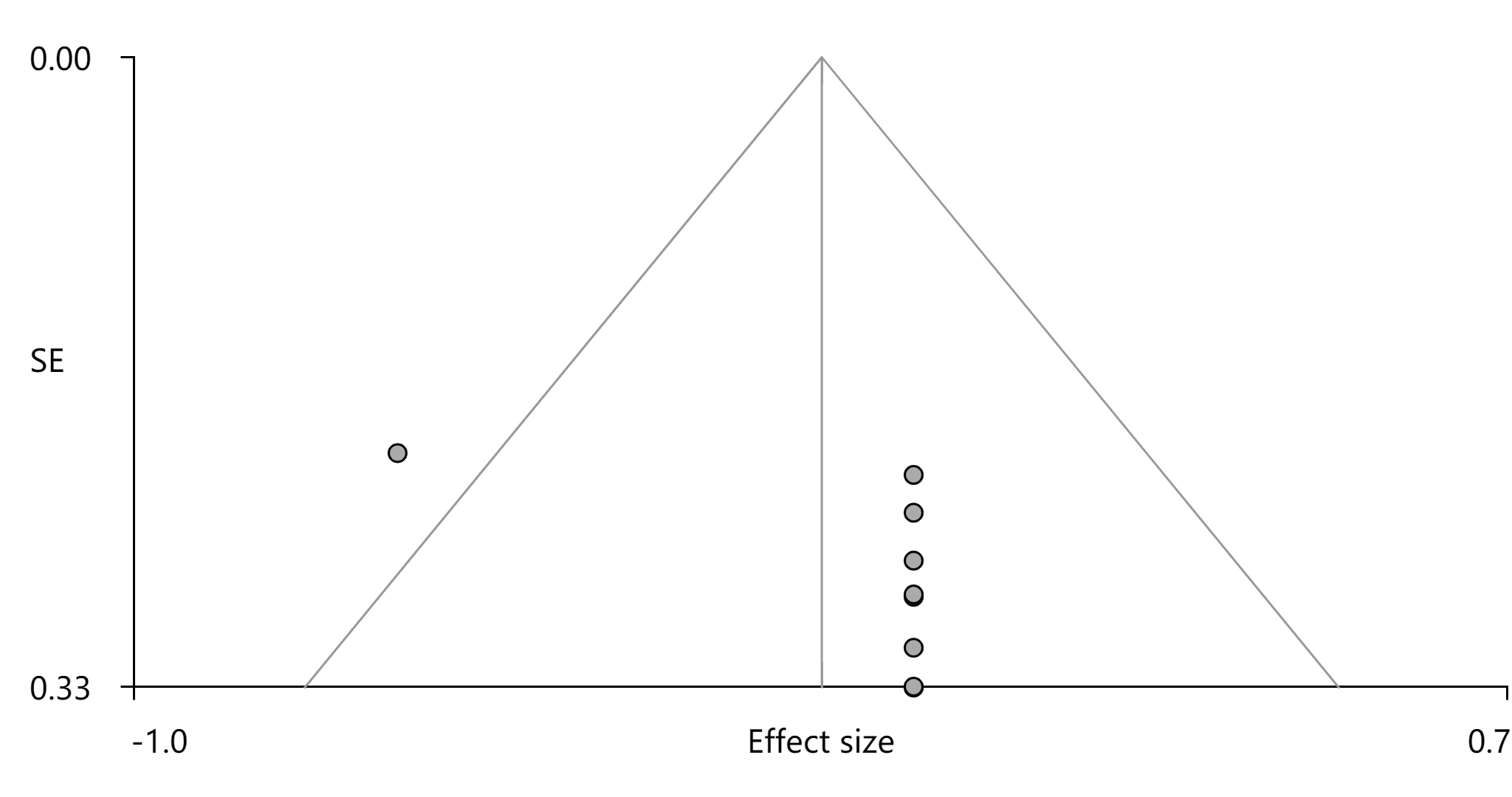

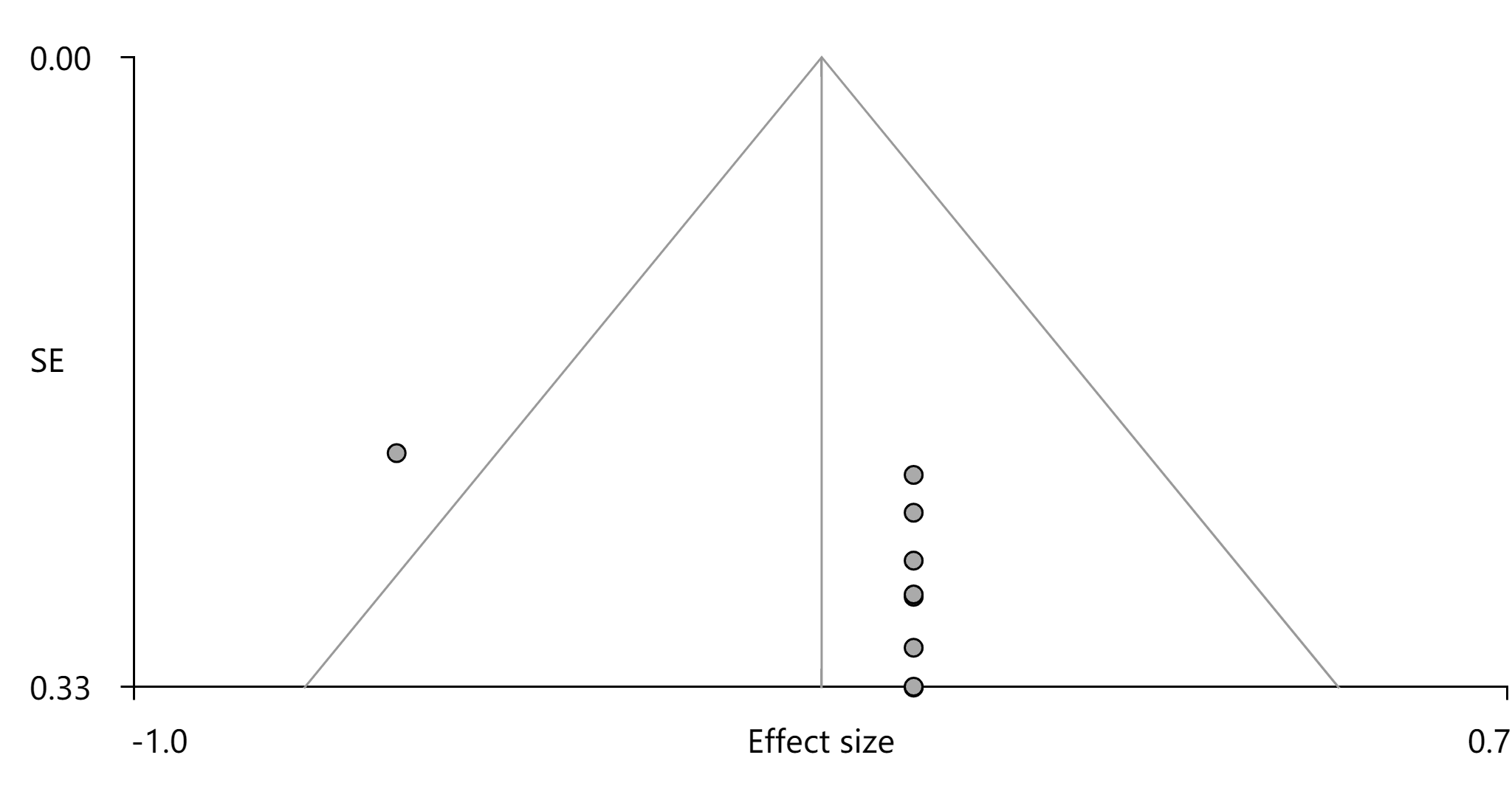

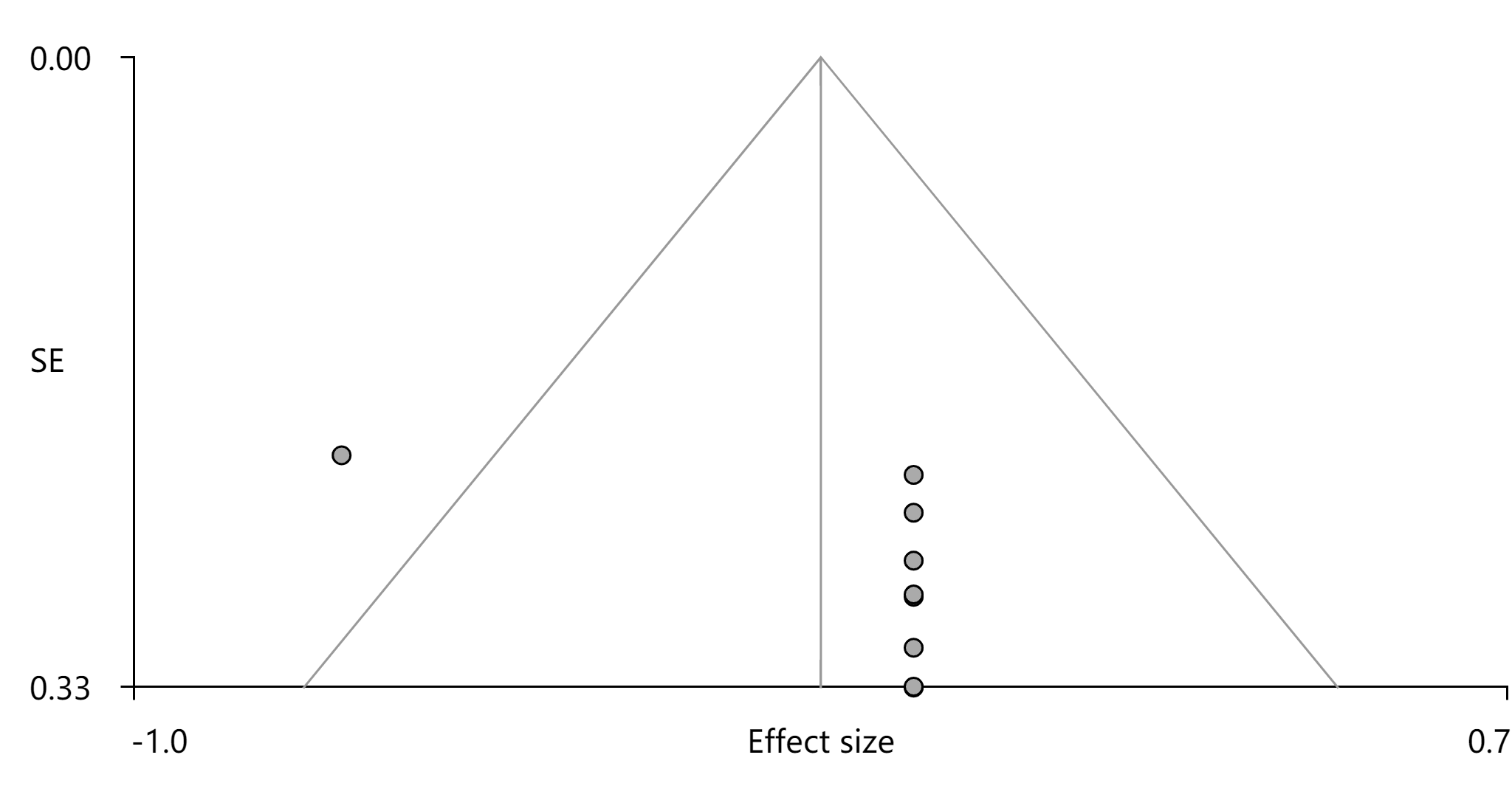

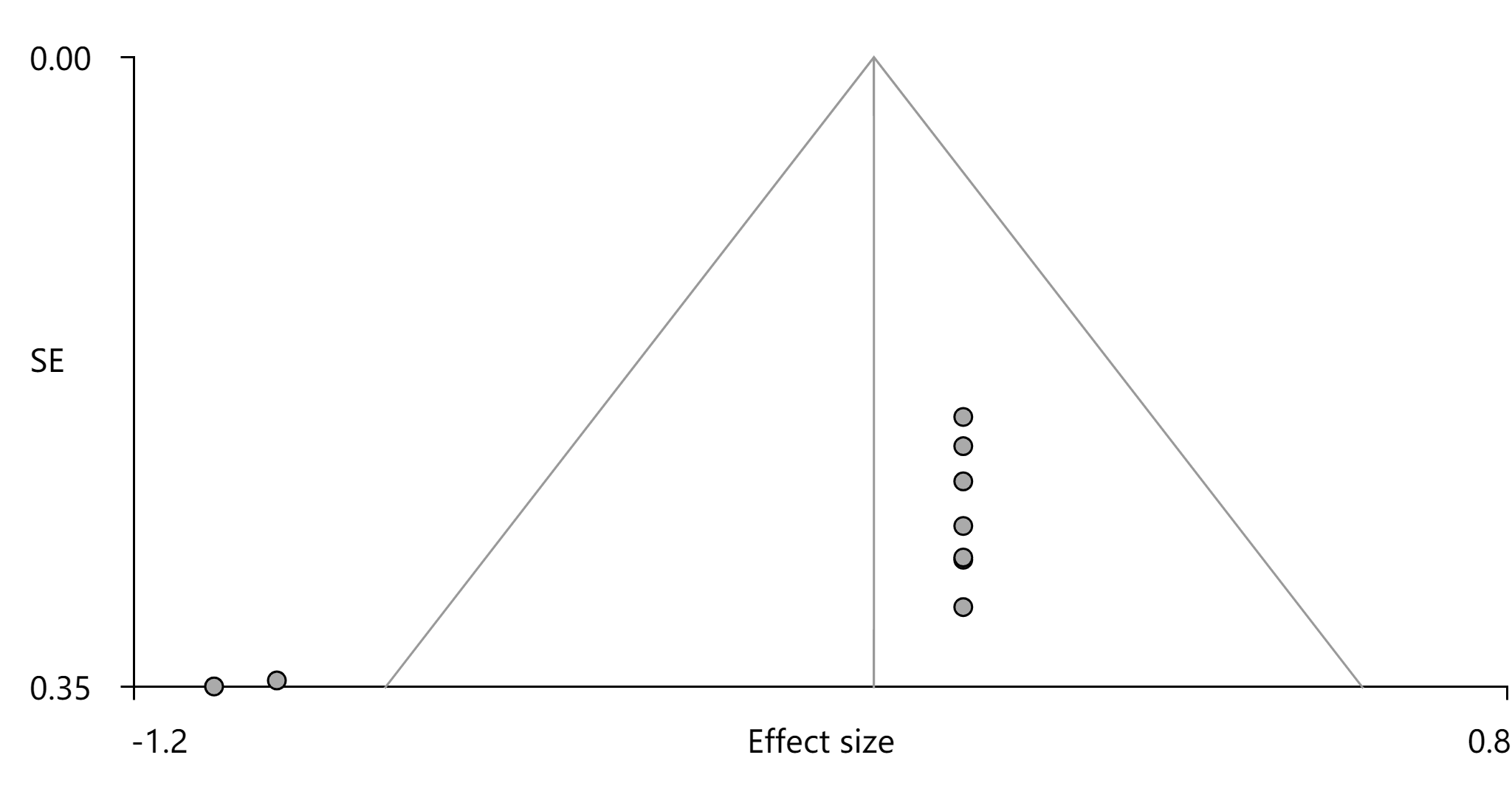
**Abbreviation:** ACC, anterior cingulate cortex; IFG, inferior frontal gyrus; INS, insula; IPG, inferior parietal gyri; L left; MTG, middle temporal gyrus; R, right; SPG, superior parietal gyrus.

（1）L ACG: Bias: -2.17, t: -0.99, df: 7, p: 0.354

（2）R STR: Bias: -2.03, t: -1.01, df: 7, p: 0.345

（3）L IFG: Bias: 2.89, t: 1.00, df: 7, p: 0.349

（4）L IPG: Bias: 3.33, t: 1.85, df: 7, p: 0.107

（5）L MTG: Bias: 3.33, t: 1.85, df: 7, p: 0.107

（6）R SPG: Bias: 3.64, t: 1.81, df: 7, p: 0.114

（7）INS.L: Bias: -5.08, t: -2.79, df: 7, p: 0.027

Figure S4 Results of funnel plot analysis for the analysis of longitudinal datasets in patients with remitted MDD.

(4) L HP Bias: 9.84, t: 4.60, df: 5, p: 0.006

(5) B MCC Bias: 7.80, t: 1.72, df: 5, p: 0.146

(6) L GR Bias: -8.15, t: -3.86, df: 5, p: 0.012


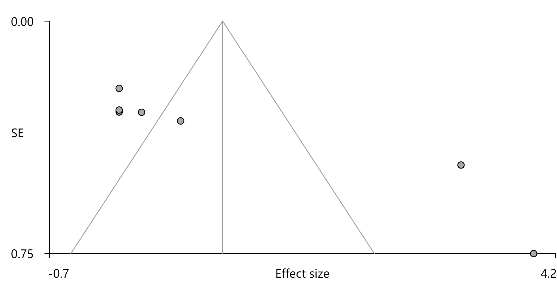


(1) L AMY Bias: 9.35, t: 4.38, df: 5, p: 0.007


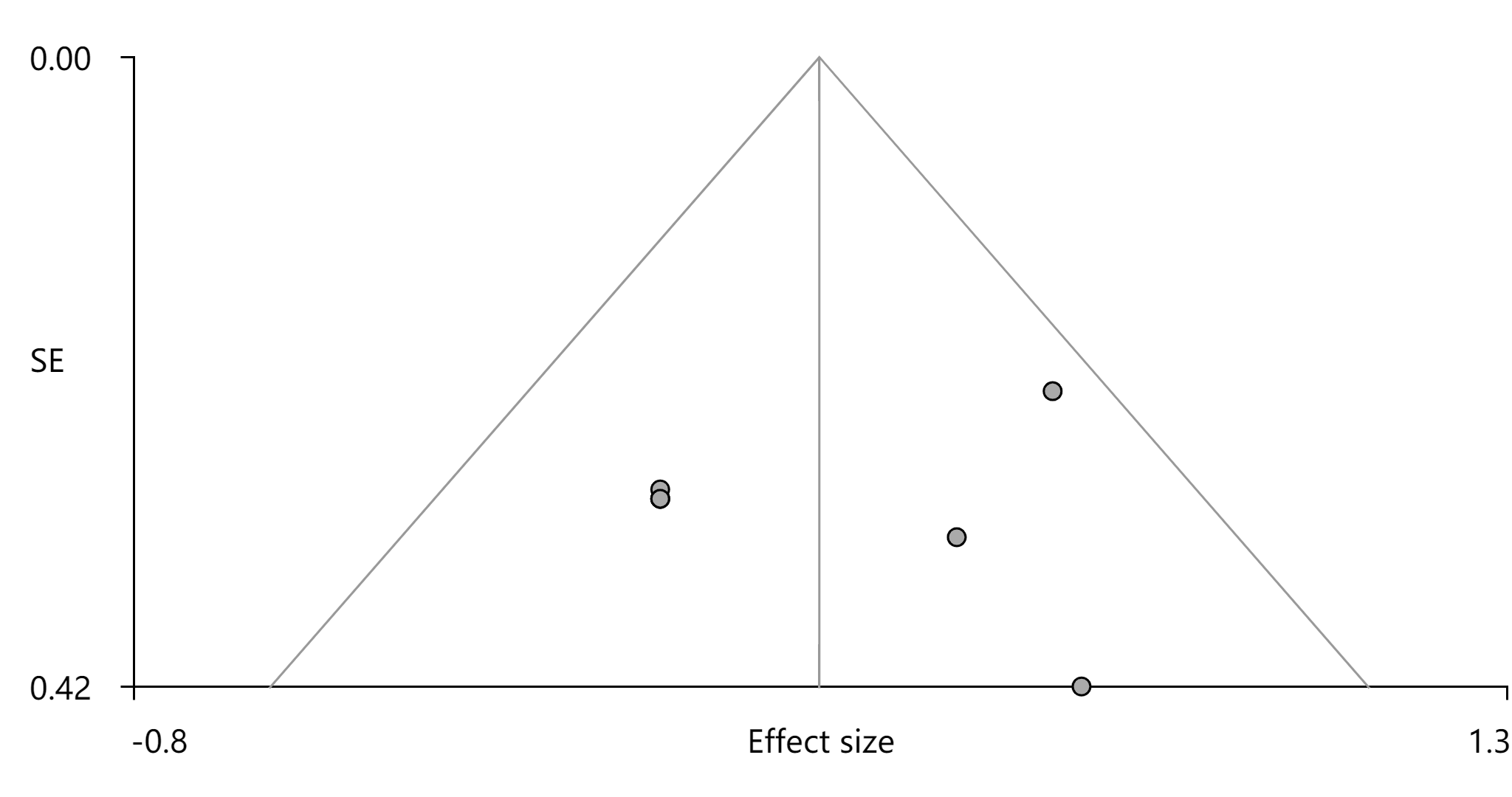


(2) L STR Bias: -0.79, t: -0.31, df: 5, p: 0.767


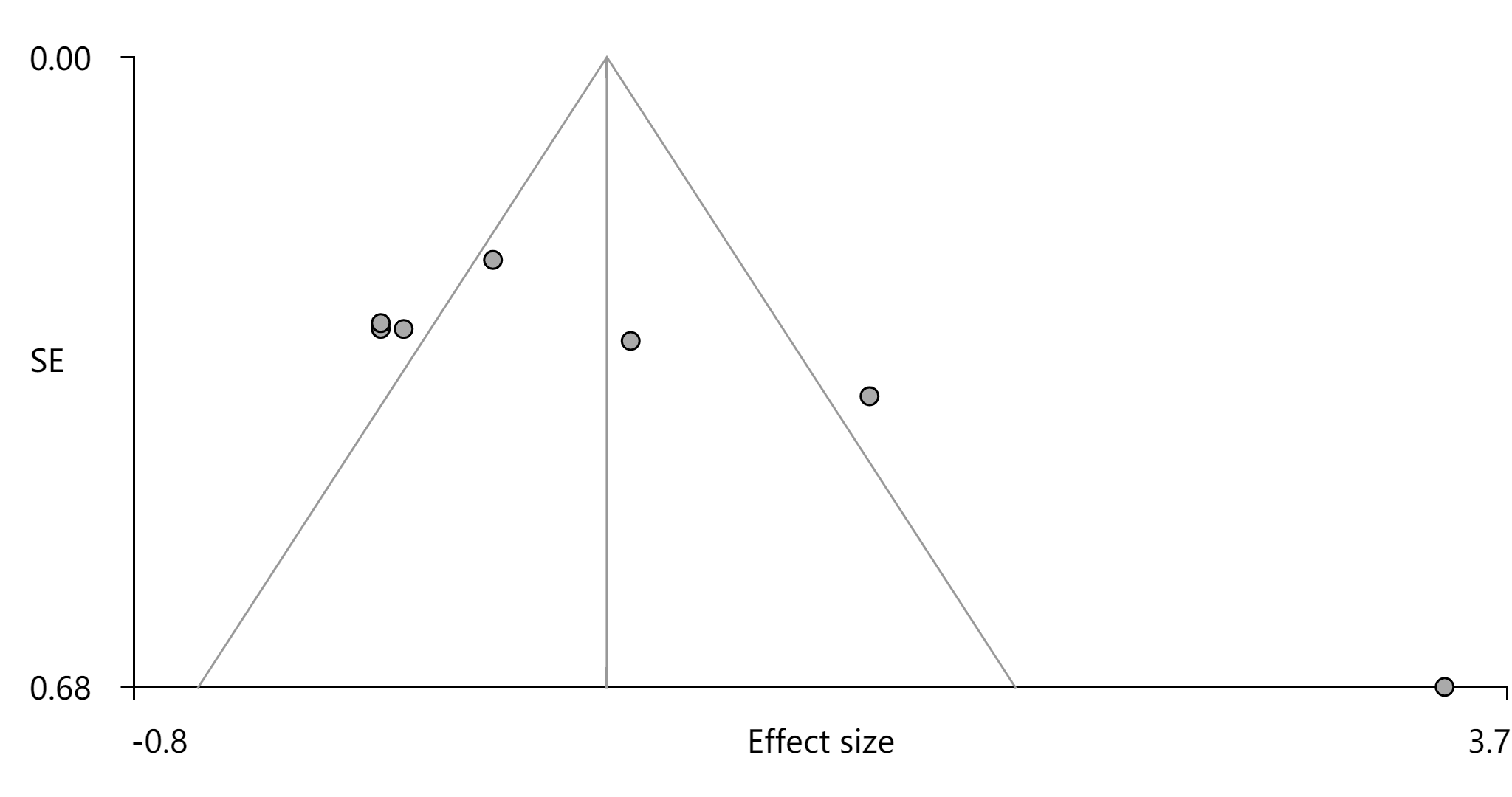


(3) L PUT Bias: 7.01, t: 2.91, df: 5, p: 0.033


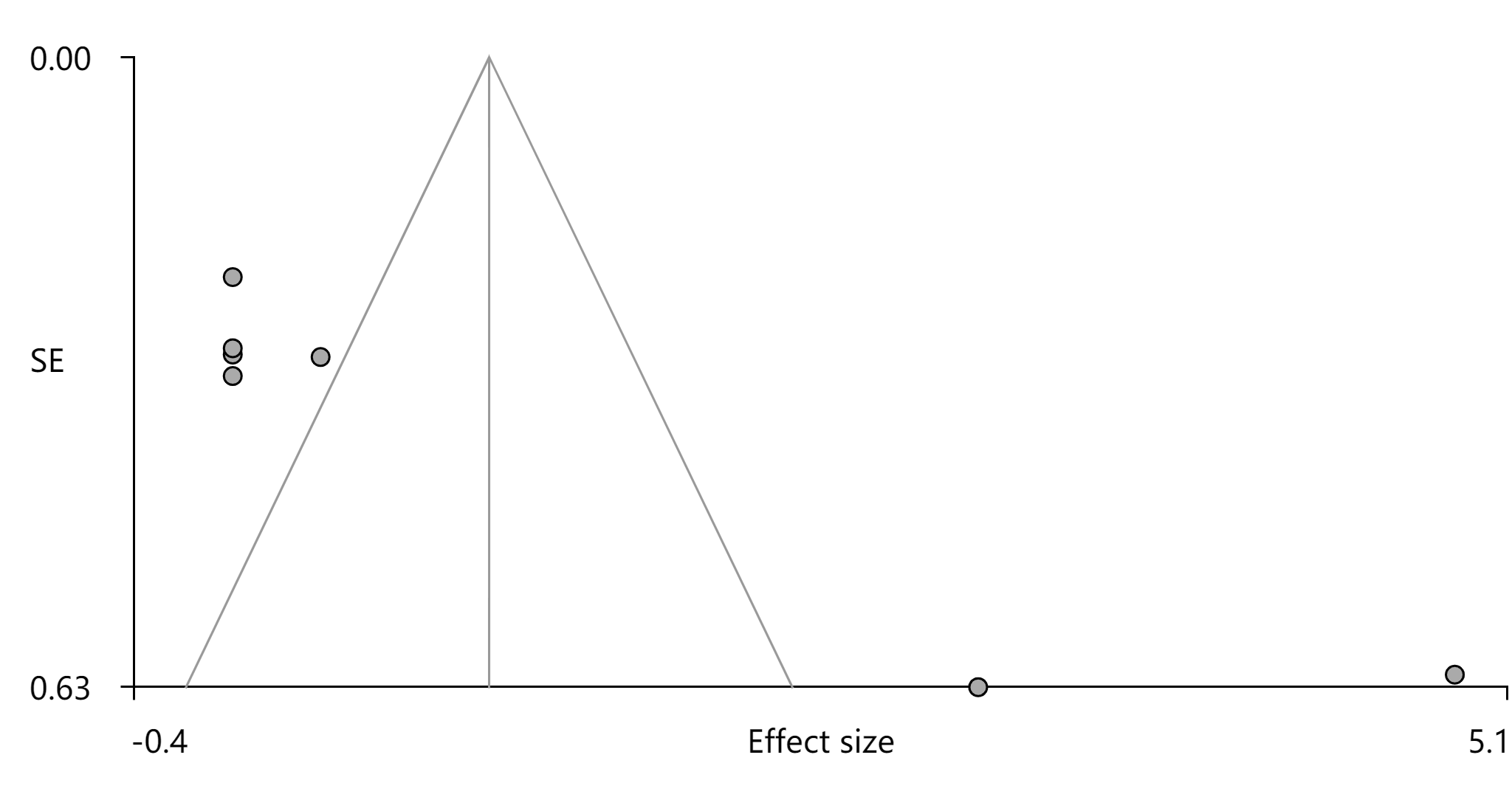

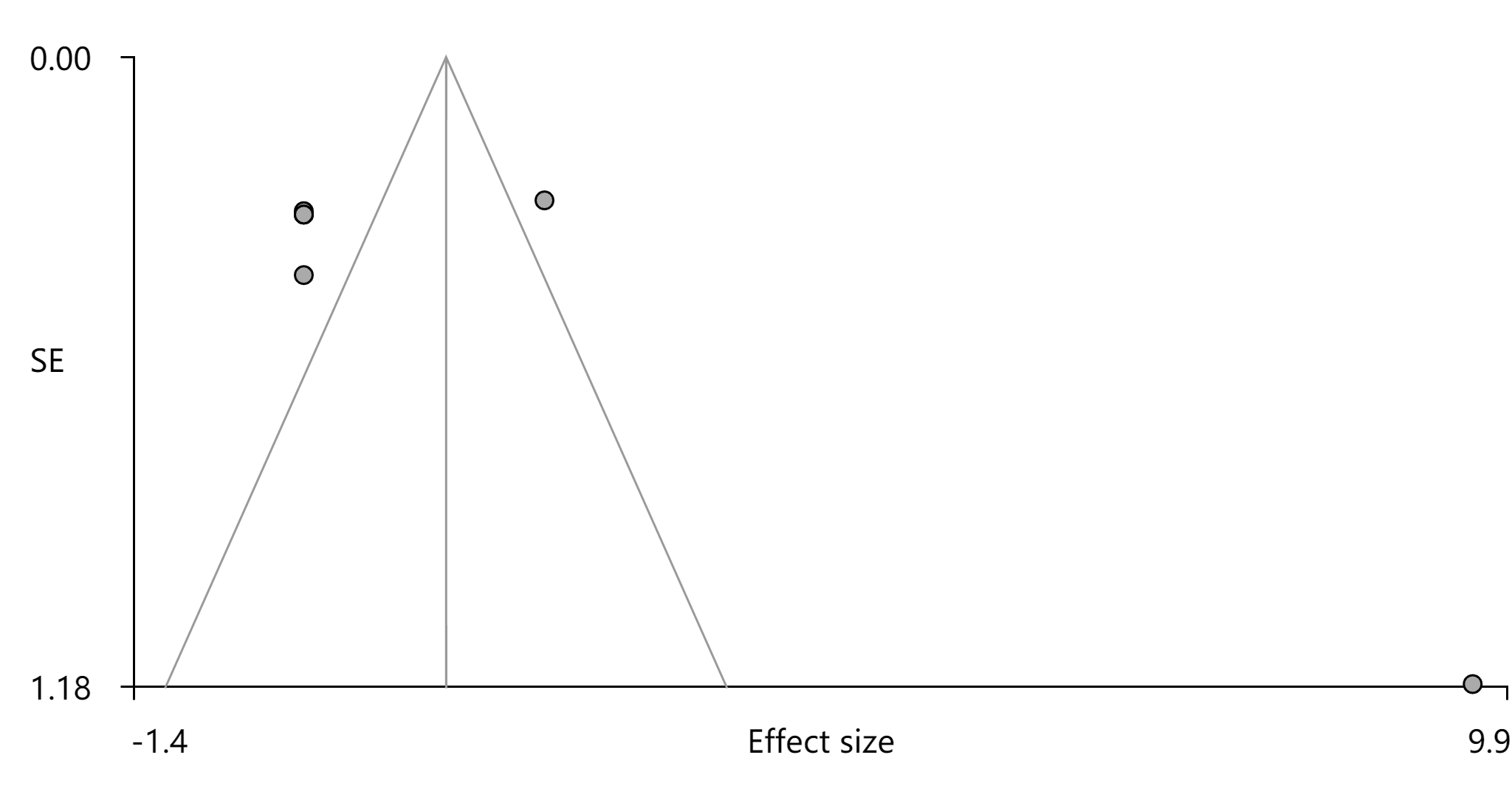

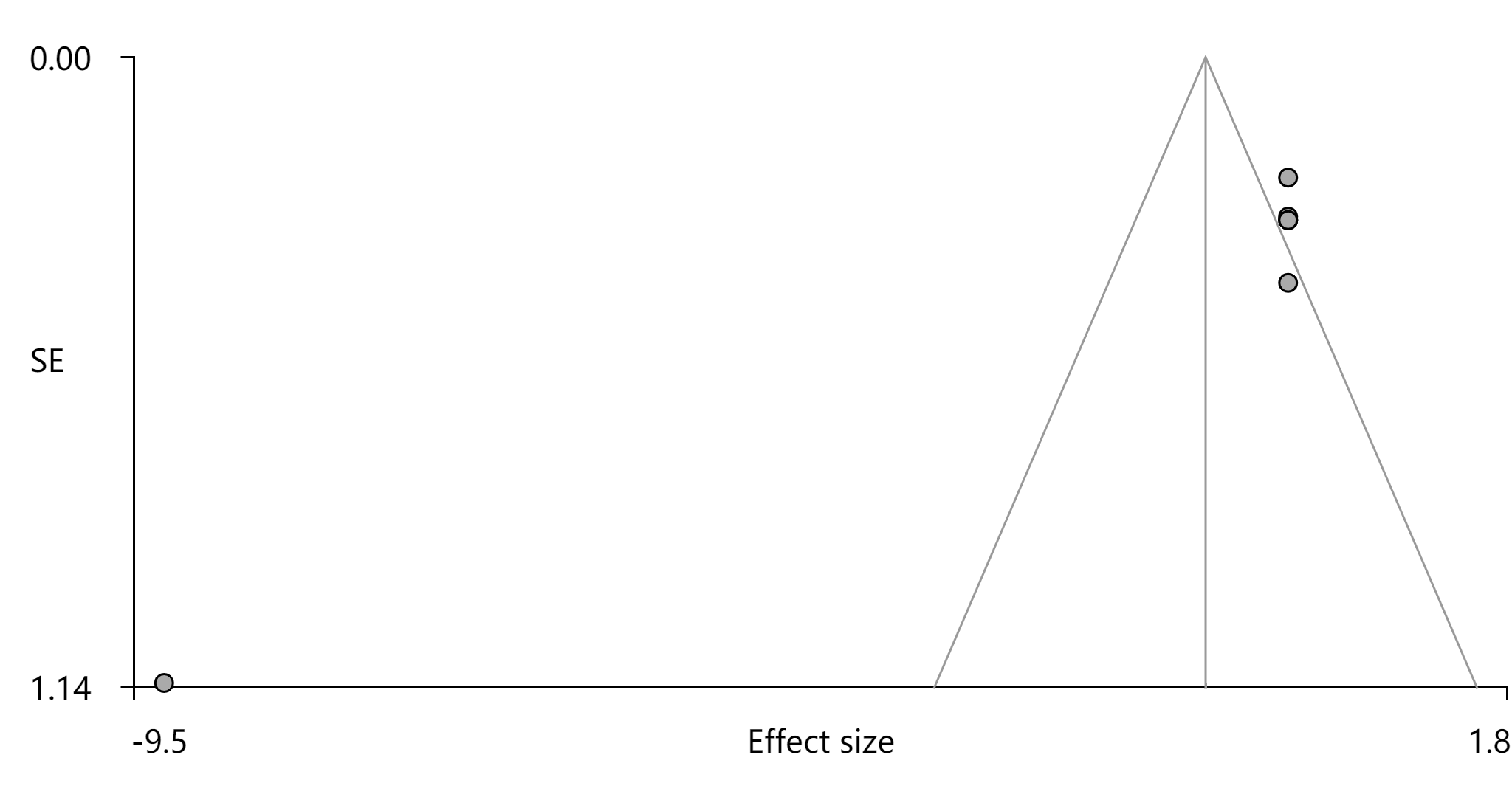


**Abbreviation:** AMY, amygdala; GR, gyrus rectus; HP, hippocampus; L left; MCC, median cingulate cortex; MDD, major depressive disorder; PUT, putamen; R, right; STR, striatum.

**Reference**

[1] Arnone D, McKie S, Elliott R, Juhasz G, Thomas EJ, Downey D, et al. State-dependent changes in hippocampal grey matter in depression. Mol Psychiatry 2013;18:1265–72. https://doi.org/10.1038/mp.2012.150.

[2] Fang J, Mao N, Jiang X, Li X, Wang B, Wang Q. Functional and Anatomical Brain Abnormalities and Effects of Antidepressant in Major Depressive Disorder: Combined Application of Voxel-Based Morphometry and Amplitude of Frequency Fluctuation in Resting State. J Comput Assist Tomogr 2015;39:766–73. https://doi.org/10.1097/RCT.0000000000000264.

[3] Klauser P, Fornito A, Lorenzetti V, Davey CG, Dwyer DB, Allen NB, et al. Cortico-limbic network abnormalities in individuals with current and past major depressive disorder. Journal of Affective Disorders 2015;173:45–52. https://doi.org/10.1016/j.jad.2014.10.041.

[4] Kong L, Wu F, Tang Y, Ren L, Kong D, Liu Y, et al. Frontal-subcortical volumetric deficits in single episode, medication-naïve depressed patients and the effects of 8 weeks fluoxetine treatment: a VBM-DARTEL study. PLoS One 2014;9:e79055. https://doi.org/10.1371/journal.pone.0079055.

[5] Lemke H, Klute H, Skupski J, Thiel K, Waltemate L, Winter A, et al. Brain structural correlates of recurrence following the first episode in patients with major depressive disorder. Transl Psychiatry 2022;12:1–9. https://doi.org/10.1038/s41398-022-02113-7.

[6] Li C-T, Lin C-P, Chou K-H, Chen I-Y, Hsieh J-C, Wu C-L, et al. Structural and cognitive deficits in remitting and non-remitting recurrent depression: a voxel-based morphometric study. Neuroimage 2010;50:347–56. <https://doi.org/10.1016/j.neuroimage.2009.11.021>.

[7] Liu C-H, Jing B, Ma X, Xu P-F, Zhang Y, Li F, et al. Voxel-based morphometry study of the insular cortex in female patients with current and remitted depression. Neuroscience 2014;262:190–9. https://doi.org/10.1016/j.neuroscience.2013.12.058.

[8] Salvadore G, Nugent AC, Lemaitre H, Luckenbaugh DA, Tinsley R, Cannon DM, et al. Prefrontal cortical abnormalities in currently depressed versus currently remitted patients with major depressive disorder. Neuroimage 2011;54:2643–51. https://doi.org/10.1016/j.neuroimage.2010.11.011.

[9] Serra-Blasco M, Portella MJ, Gómez-Ansón B, de Diego-Adeliño J, Vives-Gilabert Y, Puigdemont D, et al. Effects of illness duration and treatment resistance on grey matter abnormalities in major depression. Br J Psychiatry 2013;202:434–40. https://doi.org/10.1192/bjp.bp.112.116228.

[10] Takamiya A, Kishimoto T, Hirano J, Kikuchi T, Yamagata B, Mimura M. Association of electroconvulsive therapy-induced structural plasticity with clinical remission. Prog Neuropsychopharmacol Biol Psychiatry 2021;110:110286. https://doi.org/10.1016/j.pnpbp.2021.110286.

[11] Wang J, Wei Q, Bai T, Zhou X, Sun H, Becker B, et al. Electroconvulsive therapy selectively enhanced feedforward connectivity from fusiform face area to amygdala in major depressive disorder. Social Cognitive and Affective Neuroscience 2017;12:1983–92. https://doi.org/10.1093/scan/nsx100.

[12] Cano, M., Martínez-Zalacaín, I., Bernabéu-Sanz, Á., Contreras-Rodríguez, O., Hernández-Ribas, R., Via, E., de Arriba-Arnau, A., Gálvez, V., Urretavizcaya, M., Pujol, J., Menchón, J. M., Cardoner, N., & Soriano-Mas, C. (2017). Brain volumetric and metabolic correlates of electroconvulsive therapy for treatment-resistant depression: A longitudinal neuroimaging study. Translational Psychiatry, 7(2), e1023. https://doi.org/10.1038/tp.2016.267

[13] Zaremba, D., Dohm, K., Redlich, R., Grotegerd, D., Strojny, R., Meinert, S., Bürger, C., Enneking, V., Förster, K., Repple, J., Opel, N., Baune, B. T., Zwitserlood, P., Heindel, W., Arolt, V., Kugel, H., & Dannlowski, U. (2018). Association of Brain Cortical Changes With Relapse in Patients With Major Depressive Disorder. JAMA Psychiatry, 75(5), 484–492. https://doi.org/10.1001/jamapsychiatry.2018.0123
